# Historical Maps provide insight into a century and a half of habitat change in Fijian coasts

**DOI:** 10.1002/ece3.8153

**Published:** 2021-10-29

**Authors:** Katherine N. Lawson, Haleigh Letendre, Joshua A. Drew

**Affiliations:** ^1^ Department of Environmental and Forest Biology State University of New York College of Environmental Science and Forestry Syracuse NY USA

**Keywords:** coastal development, coral reef, GIS, historical ecology, mangrove, navigational charts

## Abstract

1. Meaningful conservation goals require setting baselines derived from long‐term ecological records and information that is rare in many regions of the world. Historical data allow us to shift baselines back in time in order to strengthen conservation outcomes in the future.
2. To explore how different histories of land use and development influenced coastal ecosystems in two Fijian cities (Suva and Savusavu), we compared a series of historical navigational charts. These charts recorded change in coral reef area and coastal mangrove forests, as well as expansions of hardened shorelines. We used geographic information systems (GIS) to georeference and make quantitative comparisons starting in 1,840 in Suva and 1876 in Savusavu.
3. Our findings show that, despite increasing urbanization in the capital Suva, available coral reef habitat has not significantly changed in over 150 years, but development has hastened a nearly 50% loss of mangroves. Meanwhile, in the smaller city of Savusavu, coral habitats suffered significant loss in area and an increase in patchiness. As in Suva, shoreline hardening increased in Savusavu, but this change was not accompanied by a loss of mangroves.
4. Nautical charts provided hitherto unavailable information on the long‐term loss and alteration of coastal habitats in Fiji. Historical ecology allows scientists to combat shifting baseline syndrome and set measured standards for conservation objectives.

## Graphical abstract

We use over 150 years of historical maps for two harbors in Fiji—including the first in‐depth navigational chart made of the region in 1,840—to chronicle the changes these coastal ecosystems, especially coral reefs and mangrove forests, have undergone through the 19th through 21st centuries. We specifically look at two harbors, the capital Suva and a smaller city Savusavu to explore how different land‐use histories have led to divergent indicators of ecosystem integrity.

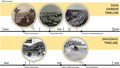

## Na uto ni tukutuku

1. Na veinaki se tatadra eso ni maroroi me vakavotukana se vakaibalebale, ena gadreva na kena veidutaitaki na veivakadidike eso me baleta nai yaubula mai na dua na gauna balavu, kei na kedrai tukutuku duidui mai na veiyasai vuravura eso. Na macala ni veivakadidike eso kei nai tukutuku eso e vakasokumuni ena gauna sa oti yani, ena vukea na kena vakaqaqacotaki ka vakavotukanataki na vakanananu ni kena maroroi se taqomaki nai yaubula duidui eso.
2. Ni railesuvi na kena vakayagataki se vakatorocaketaki na qele ena veigauna sa oti kei na kena veimatasawa e Suva kei Savusavu, e ra veidutaitaki na veitukutuku makawa eso e vakasokumuni taumada, oqo me vaka na veiveisau ni veivanua lase eso, na veidogo, kei na kena vakarabailevutaki se vakatorocaketaki na veibaravi. E vakayagataki edau nai walewale se yaragi ni veiqaravi ka vakatokai na Geographic Information Systems (GIS) me vakatauvataki na veika lelevu eso e sotavi mai na yabaki 1840 I Suva kei na na yabaki 1876 mai Savusavu.
3. E dina ni vakatakila na macala ni vakadidike, na tubu cake ni bula ena koro turaga e Suva, ia na veicakau e sega ni veisau ena loma ni 150 na yabaki, ia na revurevu ni veivakatorocaketaki e kovuta ni rauta e 50% ni veidogo. E laurai ni levu sara na kena vakacacani na veicakau mai na tauni i Savusavu ni vakatauvatani kei Suva. E laurai ni sega ni vu mai na veika oqo ena kena vakacacani na veidogo.
4. Na mape era vakarautaki ena vuku ni veimataqali tukutuku era solia, e laurai ni gauna e vakayacori kina na veitalanoa o ya, ka yacova mai nikua, e se sega ga ni kunei se cava e vakavuna na kena yali se veisau nai tuvaki ni veimatasawa e Viti. Na noda veimaliwai na tamata kei na veikabula eda bula veitikivi ena dua na gauna balavu ena vukei ira na daunivakadidike ena kena valuti na veika e vakatokai na Shifting Baseline Syndrome me rawa ni vakadeitaki nai walewale ni se i naki ni kena maroroi se vakayagataki vakamatau na nodai yaubula eso.

## 1 INTRODUCTION

Coral reefs and mangrove forests face numerous threats that are leading to levels of unprecedented change in the Holocene, including those from climate change (Spalding & Brown, 2015), increased storm frequency and intensity (Cheal et al., 2017), unsustainable fishing practices (Rhodes et al., 2018), and deleterious land management practices (Shuler et al., 2019). Preserving biodiversity on coral reefs and mangrove ecosystems are high priorities for conservation (Duke et al., 2007; Knowlton, 2001; Munday, 2004; Pratchett et al., 2011). What is perhaps most challenging about these losses is not only the rapidity with which they have taken place but also the speed at which altered states have been accepted as the new baseline (Klein & Thurston, 2016). Under the paradigm of “Shifting Baseline Syndrome” (Pauly, 1995), each successive generation defines a system's natural state as the conditions that exist when they are first exposed to it. Absent information to the contrary, environmental change that occurred before that first experience is rendered invisible. This process perpetuates intergenerational amnesia about what makes “natural” and “healthy” ecosystems.

Loss of historical perspective on environmental change presents a challenge to management and conservation because it represents a diminishment of the natural world and an acquiescence to environmental degradation. Without adequate baselines, how can we establish meaningful management goals? Thus, a critical step for environmental managers and conservation biologists is defining the tempo and modes of environmental change. However, one challenge facing managers, particularly those working in the tropical Pacific, is a lack of long‐term data. These kinds of data are essential in combating shifting baseline syndrome because they transcend the living memories of current populations and recall a world that no one now remembers. Without these records that world, and the conservation and restoration potential it represents, passes from living memory and out of our collective recognition.

One potentially useful source of long‐term data is geospatial data from aerial photography and satellite imagery (Palandro et al., 2008; Whipple et al., 2011). Empirical, quantifiable data from these images can be analyzed with geographic information systems (GIS) to provide a continuity of records. However, most aerial photography only goes back 60–70 years and the first LANDSAT satellite was launched in 1972. Hence, their ability to push back against shifted baselines is limited (Bromberg & Bertness, 2005). Documenting change prior to photographs and satellite imagery requires exploring other sources.

Historical maps and charts offer the possibility of gathering data that predate aerial and satellite photography. This is especially true for navigational charts that pay special attention to the location and extent of marine hazards, including coral reefs, and shoreline features, like mangrove forests. Historical charts have been used to show changes in reef coverage in the Florida Keys over a 240‐year period (McClenachan et al., 2017), kelp forests in British Columbia for nearly a century (Costa et al., 2020), and maps have been merged with aerial photography to chronicle changes in California oak savannahs for over a century (Whipple et al., 2011). Navigational charts in the South Pacific, created by Europeans and Americans, provide opportunities to explore ecosystem changes in places lacking other published records. Reefs and mangrove forests are especially important in Fiji, where the Indigenous community remains deeply connected to marine habitats.

### 1.1 *Na* *kena mapetaki na loma ni vanua kei na wasawasa—*Mapping the land and the sea

The Republic of Fiji is an archipelago in the South Pacific. Native Fijians (*iTaukei*) have been in Fiji for at least 3,500 years and continue to have close associations with the land (*vanua*) and sea (*waitui*). The archipelago has been a center for economic and social trade and mobility for millennia (Cochrane, 2017). While both Tasman (in 1643) and Cook (in 1784) sighted the islands, the first European to make a comprehensive map of Fiji was William Bligh, who after mutiny on the H.M.S. Bounty sailed through the Fijian archipelago en route to what is now Indonesia (Bligh, 2013). Additional charts were made by the United States Exploring Expedition in 1,840 (Wilkes, 1845, Figure 1) in support of burgeoning U.S. colonial interests (Smith, 2013). By the time the United Kingdom seized control of Indigenous lands in 1874, the British Admiralty was already publishing charts to help commercial vessels navigate the reefs. Suva Harbor became Fiji's capital in 1882 (Seemann, 1862). Suva's conjoined political and economic valuation led to a rapid population increase in the city supplemented by a trend toward increasing urbanization during the mid‐20th century (Ward, 1959).

**FIGURE 1:**
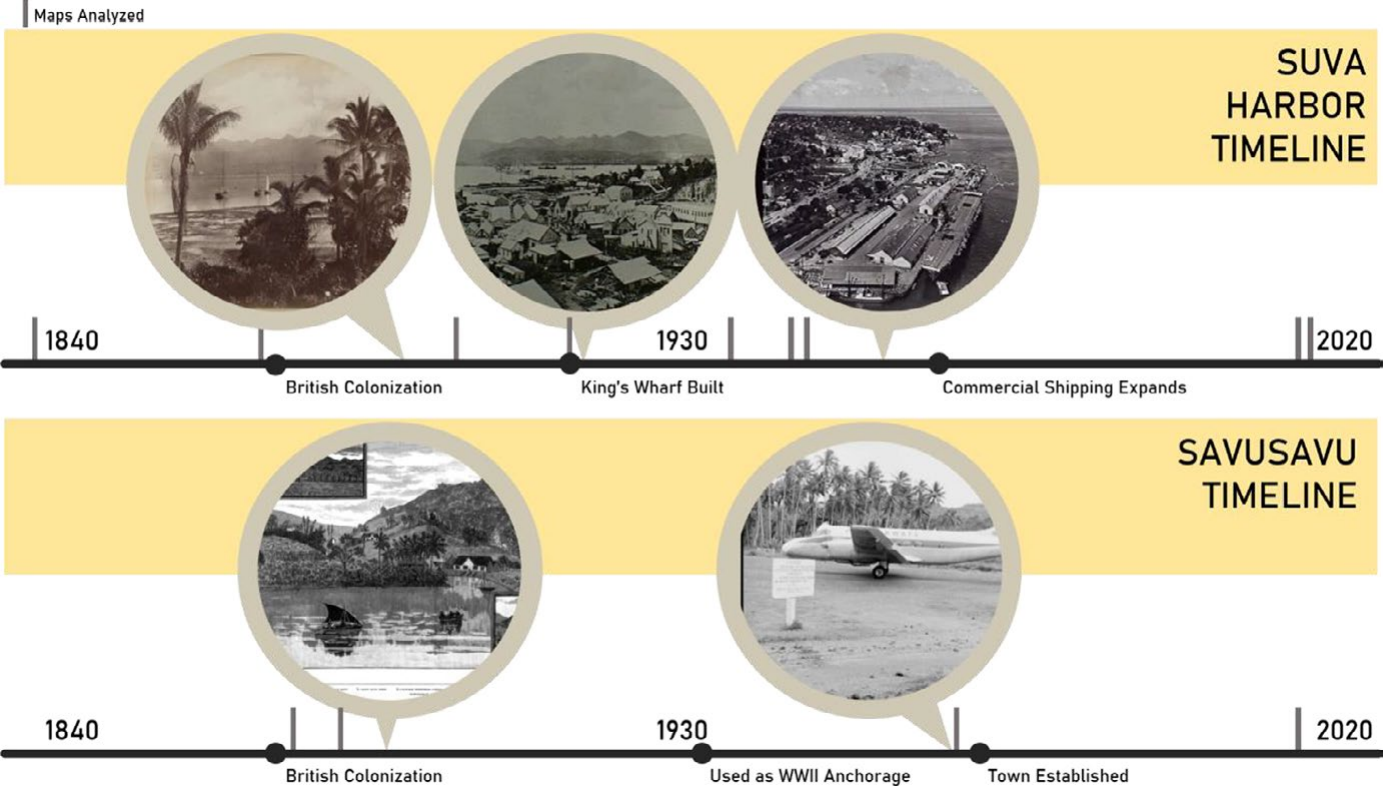
Timeline of relevant historical events occurring within the time analyzed in this study. Top row left to right, historical photographs show Suva Harbor in 1890 (Charles Kerry, National Library of Australia), 1914 (Australian War Museum), and 1956 (Australian War Museum) and bottom row, left to right shows a postcard of Savusavu in 1888 (State Library Victoria) and a photograph from 1966 (Michael Terry, National Library of Australia). Gray dashes show charts analyzed in this study. See Supporting Information for more photograph source information

In the early 1900s, Suva had an array of marine habitats including corals and mangroves (Sparling, 1923). Davis (1918) described the reefs of Suva thusly: “Among other associates of the lagoon corals are sea urchins with long black spines like knitting needles, large dark blue starfish, slowly crawling reddish ‐brown sea ‐ cucumbers, and many kinds of fish of varied hues … which swim about at ease among the coral branches. … The most remarkable are the gigantic clams, one or two feet in length, which live embedded in the reef flat, hinge‐line down…” Some twenty years later Barrett (1935) opined, “The crystal‐clear waters of the lagoons of Fiji Harbour contain many bright‐hued fish and coral formations of a fairy‐like beauty.”

Today, about a third of the country's population lives within or near Suva (Dacks et al., 2020), which has led to declining environmental conditions. By the end of the 20th century the harbor had been dredged (Penn, 1983), shipyards were polluting local fisheries (Davis et al., 1999) and relic reefs were subject to periodic inundation of sewage (Lal et al., 2018).

While Suva was experiencing these degradations, Savusavu on the northern island of Vanua Levu was experiencing a different suite of human interactions. The town of Savusavu was established in 1969, although geothermal activity attracted small settlements much earlier (Dana, 1849). James Dwight Dana (1849) describes the springs around Savusavu, “A plain rising with a gentle slope from the water extends back from the shore about three‐fourths of a mile, and then passes into the steep declivities of a high broken ridge, running nearly parallel with the coast. Patches of grass, and much low, dense shrubbery, cover the plain near the springs; and some distance to the right and left, large coconut groves throw some little beauty into a scene otherwise unattractive.”

In addition to hot springs and coconut groves, Savusavu boasts a natural deep harbor—one that was so large that it was considered an ideal spot to shelter the U.S. Pacific fleet during World War II (Inskip, 1937). While development around Savusavu has mostly centered on dried coconut (copra) production, in more recent times, tourism has emerged as a larger economic driver (Graci & Vliet, 2020). Along with this shift has been a conversion of mangroves to beachfront (J. Drew, personal observation) and other structural and environmental changes including proposed geothermal and solar activities (Prasad & Raturi, 2019). Thus, in comparison to Suva, shoreline development occurred without major shipping and population pressures. Therefore, understanding the history of Savusavu gives us insight into the ways human activity around smaller settlements may have affected coastal morphology and biodiversity.

We aim to use historical charts to track how coral reefs and associated ecosystems in Suva and Savusavu have changed over time. Many *iTaukei* rely on mangrove and coral habitat for food, income, and ecosystem services (O’Garra, 2009; Sangha et al., 2019) and identifying major habitat losses could shape conservation efforts in the region by changing our understanding of what these ecosystems look like when healthy. We specifically use the following metrics to evaluate these system indicators: coral cover, coral fragmentation, mangrove extent, and shoreline hardening. Together these indicators allow us to assess how coastal habitats off these two Fijian cities have changed over the past 160 years. As Suva has seen major industrialization, and other studies have recorded reef degradation (e.g., Lal et al., 2018), we expected to see both loss of overall reef area and increased reef edges in the later years of our study. In Savusavu, which has not experienced a similar increase in population, we expected to see less reef loss. At both sites, we expected a decline in mangroves, as there has been extensive loss of forests in Fiji (Lal, 1990). By setting earlier baselines from which to evaluate current and future conservation and management plans, our work improves conservation goals by resetting these shifted baselines (Bao & Drew, 2017) and by helping people remember the vivid splendor of Fijian reefs.

## 2 METHODS

**FIGURE 2:**
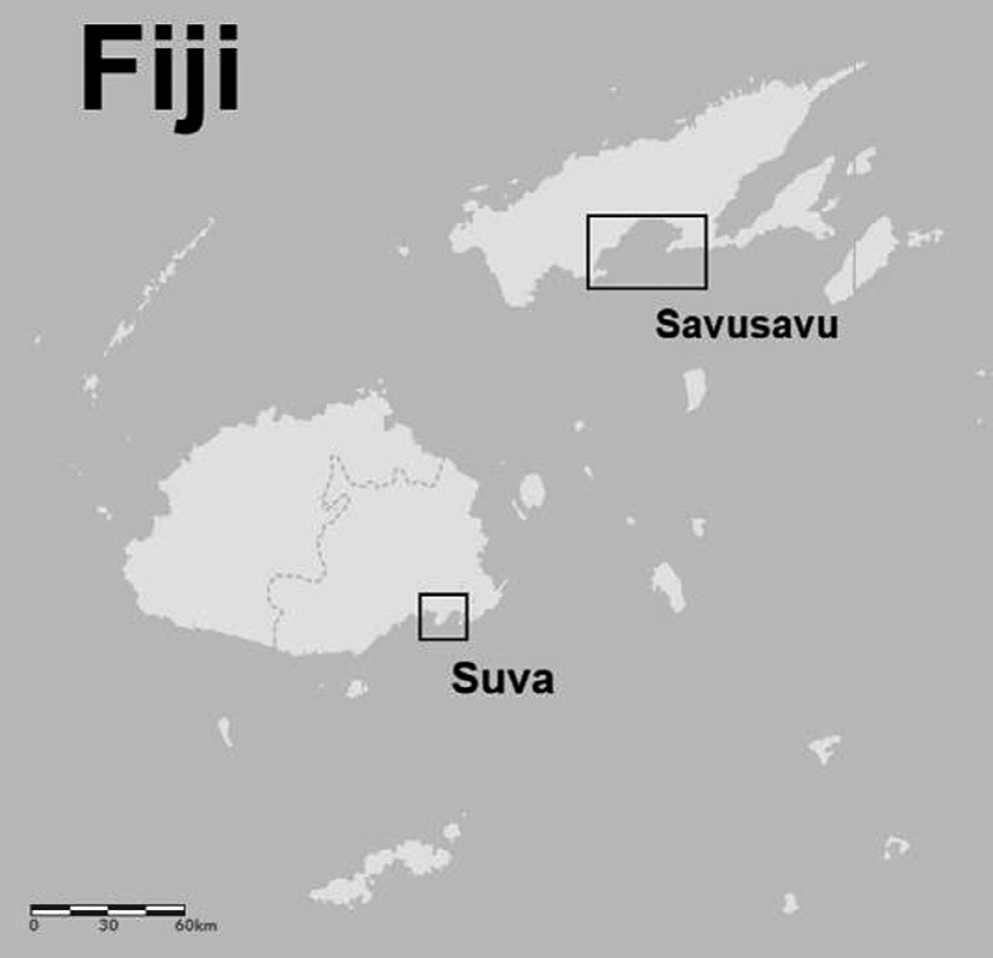
Locations of Suva and Savusavu in Fiji, with boxes showing the approximate extent of charts used in this project

We reviewed over 150 years of change in recorded coral and mangrove habitat from Suva Harbor and Savusavu (Figure 2). We examined scans of historical charts from the British Admiralty, U.S. Exploring Expedition, U.S. Army, and Fijian Lands and Survey Department. Charts were acquired from the U.K. Hydrographic Office, National Library of Australia, State Library of New South Wales, and the Bodleian Library at Oxford University. Initial searches found over 50 charts that were identified for possible use in this project. The charts chosen for analysis do not represent the only ones available in the area, but all the available charts with sufficient coral reef or mangrove detail and comparable scaling. Earlier and later charts are highly detailed and best represent the change in coral area.

To the best of our knowledge, individual charts represent separate surveying events (sampling efforts) or reflect major edits of earlier charts based on new survey data. Charts that were based on the same sampling events were excluded from analysis to avoid replication and undue correlation. These decisions were based on data provided on the charts themselves. Publication dates are the years used in of analysis, as these represent the latest point of review.

ArcGIS Desktop 10.8.0 and R Studio version 1.3.1073 were used to prepare charts for analysis. Historical chart scans were georeferenced onto WGS 1984 following reference points procedure outlined in McClenachan et al. (2017) and Costa et al. (2020). At least four referenced points were used per chart. A Projective and First‐Order Polynomial Transformation were compared for best fit and selected based on lowest RMS error (each map <0.001 RMS error). Charts compared in this analysis cover a relatively small geographic area and were georeferenced with low RMS error, minimizing the possible geodetic or planimetric error (Jenny & Hurni, 2011).

**FIGURE 3:**
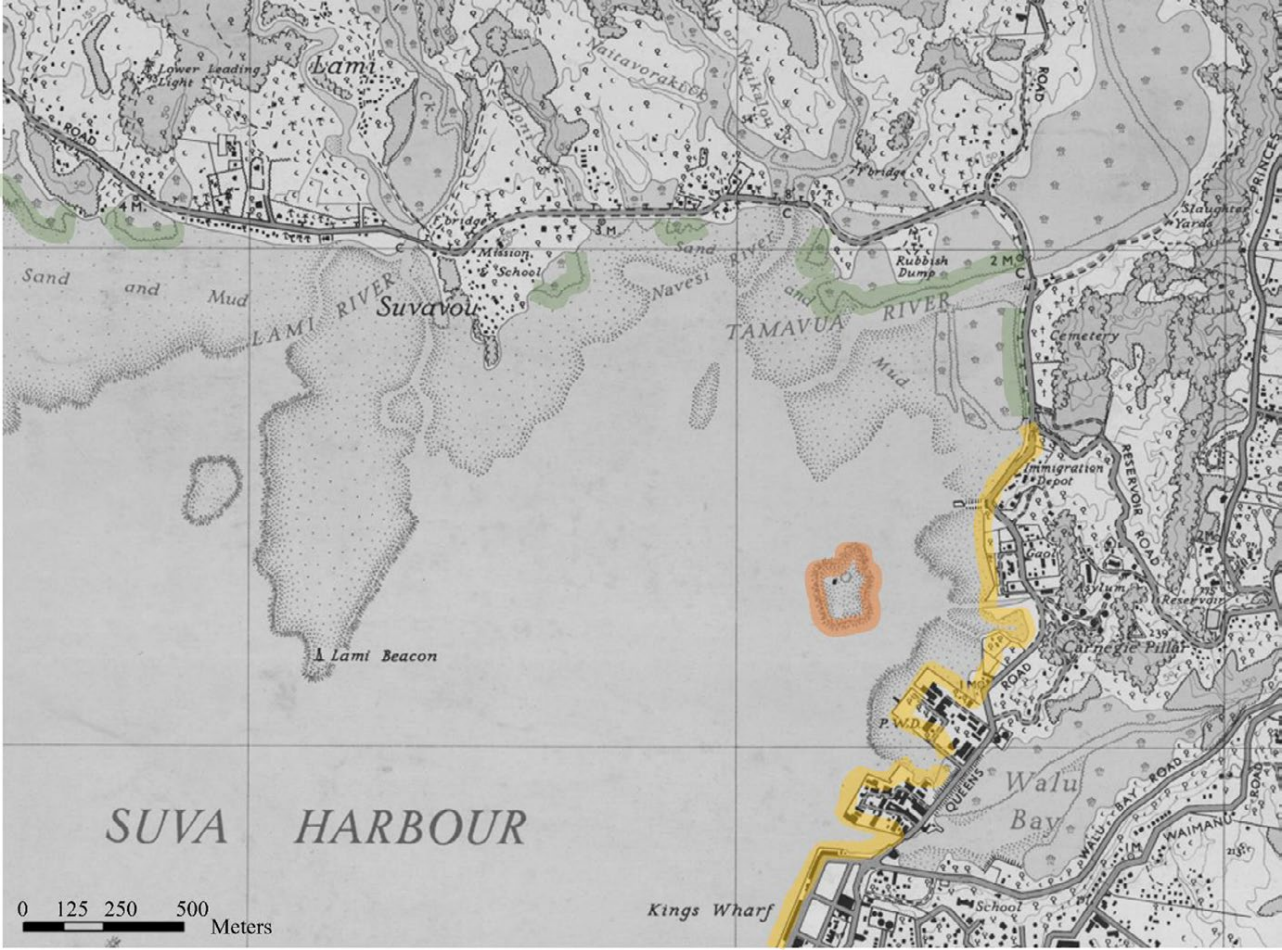
Clipping of a 1945 Suva chart, acquired from State Library of New South Wales (Table 1). Color of original chart has been edited to grayscale to highlight features of interest. In clipping, coral is represented by hatched coastline on the original chart and highlighted in orange, mangrove is represented by tree symbols and curled lines on the original and highlighted in green, while hardened shorelines is represented by dark gray lines and shapes and highlighted in yellow

We measured change in habitat and other landscape metrics by creating polygon features of coral reefs and polyline features of mangrove coastline habitat. We used editor tools to manually vectorize features from historical charts through heads‐up digitizing. Charts drawn before 2018 included hand‐drawn descriptions of coral and mangrove habitat. Coral reefs were identified based on written labels, symbols, and changes in soundings recorded on the charts (Figure 3). In a few cases where cartographers differed in the substrate designation of large patches, editorial decisions were made to keep charts comparable. Polygon vectors were transformed to raster layers with ArcMap Conversion tools. A raster cell size (17.32 m) was chosen to account for differences in map scale and the detail drawn upon them and is equal to the smallest charted reef (300 m^2^). Spatial Analyst tools were used to reclassify polygons and background area to coral and noncoral habitat. For each year and location, we compared change in coral reef habitat in terms of total area, number of patches, and edge length through time. We used the tools CA (patch area), CORE (core area, or the area of each patch which is not edge), NP (number of patches), PD (patch density, or the number of patches per the total area, standardized for comparison), PERIM (length of edge), and ED (edge density, or the total edge length per the total area, standardized for comparison) from R package landscapemetrics (Hesselbarth et al., 2019) to compare raster metrics.

Mangrove forests were identified from labels, symbols, and coastline features. Change in mangrove habitat was compared as a proportion of total coastline length. Shoreline hardening, where natural coastal interfaces such as mangroves or sand shores are replaced by human‐built surfaces, was measured in terms of the proportion of total coastline where anthropogenic structures (including buildings, docks, roads, naval bases, golf courses, and rifle ranges) met the coastline and no mangroves were present. These structures were depicted on the charts with varying line thickness, shapes, dashes, etc. and labels. River mouths were excluded from coastline measurements. Nonmangrove and nonanthropogenic coastline was categorized as other, which included meadows, nonmangrove tree stands, beaches, rocky shore, and other natural habitats. Measurements were calculated as proportions of total coastline to account for differences in coastline detail among charts.

To accurately compare coral and mangrove habitat among charts with different levels of detail, extent, and scale, not all charts are used in every analysis (Table 1). Coral and mangrove habitat was compared in overlapping areas so as not to misrepresent gain or loss. Suva charts showed the same extent per each time period; however, charts for Savusavu depicted a much greater area than Suva charts, so two separate analyses were completed. The first focused on Savusavu town and surrounding reefs, so that a similar study area (~3,625 ha) could be compared with Suva. The second utilized all data available for Savusavu Bay, an area of roughly 112,000 ha. Despite the disparity in extent between maps of Suva and Savusavu, roughly similar levels of detail are available for each location. For the 1966 time period in Savusavu, two contemporary charts from the same source were merged to create a single layer. The western portion was published in 1962, the east in 1966.

**TABLE 1:** Historical charts of Suva and Savusavu Harbor used in this manuscript, including citation information and analyses included. Note the scale for 1840 Suva is an approximation as true scale is not provided on the chart

Title and author	Date	Scale	Publisher/creator	Library, archive, source of reproduction	Coral area analysis	Mangrove length analysis
Suva Harbor						
Suva Harbor south side of Viti Levu, 1,840, C. Wilkes	1840	Approximately 1:50,000	U.S. Exploring Expedition	State Library New South Wales	Graphed, not analyzed	N/A
Fiji Islands: Viti Levu: Nukulau Island to Namuka Island Including Lauthala, Suva, and Namuka Bays, L.S. Dawson	1875	1:24,370	UK Hydrographic Office	Bodleian Library at Oxford	Included in analysis	N/A
Fiji Islands: Viti Levu: Suva Harbor (Tomba Ko Suva), J.W. Combe	1898	1:12,150	UK Hydrographic Office	UK Hydrographic Office	Included in analysis	Included in analysis
Fiji Islands: Viti Levu: Nukulau Island to Namuka Island Including Lauthala, Suva and Namuka Harbs. New Edition Dec. 1914. L.S. Dawson	1914	1:24,370	UK Hydrographic Office	Bodleian Library at Oxford	Not analyzed	Included in analysis
Fiji, Viti Levu, Suva & Lower Rewa, Tourism, 1934, 1:63,360	1934	1:63,360	Fiji Lands and Survey Department	Australian National Library	Included in analysis	Included in analysis
Fiji, Viti Levu, Suva, Series: X751, Sheet 67, 1943, 1:62,500	1943	1:62,500	U.S. Army Map Service	Australian National Library	N/A	Included in analysis
Fiji—Suva [cartographic material]/published (with the help of various New Zealand agencies) by the New Zealand Department of Lands and Surveys	1945	1:12,672	Fiji Lands and Survey Department, New Zealand Department of Lands and Survey	State Library New South Wales	Included in analysis	Included in analysis
Fiji Islands: Viti Levu: Suva Harbor	2019	1:12,000	UK Hydrographic Office	UK Hydrographic Office	Included in analysis	Included in analysis
Savusavu Bay						
Fiji Islands: Vanua Levu: Savu Savu Bay, H. Barrack	1876	1:177,100	UK Hydrographic Office	Bodleian Library at Oxford	Graphed, not analyzed	N/A
Fiji Islands: Vanua Levu: Savusavu Bay, United States Hydrographic Office, 3rd edition, No. 2853, 1921	1,880	1:100,920	UK Hydrographic Office	U.C. San Diego Library	Included in analysis	Included in analysis
Fiji, Vanua Levu, Savusavu Bay West, Series: X754, Sheet 11, 1962, 1:50,000, & Fiji, Vanua Levu, Savusavu Bay East, Series: X754, Sheet 12, 1966, 1:50,000	1966*	1:50,000 (over 2 charts)	Directorate of Overseas Surveys	Australian National Library	Included in analysis	Included in analysis
Fiji Islands: Vanua Levu: Savusavu Bay	2018	1:50,000	UK Hydrographic Office	UK Hydrographic Office	Included in analysis	Included in analysis

Landscape metrics were compared for coral reefs at both sites with chi‐square tests. In Suva, the proportion of mangrove coastline was statistically compared among years with a linear model. Normality was confirmed with a Shapiro–Wilk Test prior to modeling (“stats” R Core Team, 2020). In Savusavu, three years of available mangrove data was statistically compared with a chi‐square test, due to the small sample size.

## 3 RESULTS

We located eight charts from Suva and four charts for Savusavu covering nearly 180 and 150 years, respectively (Table 1). Seven charts had sufficient coral detail, but the 1914 Suva chart depicted coral from a sampling event that was used in 1898, so the 1914 map was excluded from analysis. The oldest charts found for both sites depicted the least amount of coral detail, or topographic accuracy (see Lukas, 2014), likely reflecting the cartographic capability at that time rather than true differences, and the earliest Suva map had poor planimetric accuracy, or fidelity to current maps through georeferencing (see Lukas, 2014). To be conservative, statistical analysis of coral change started at the next earliest time point, running from 1875 to 2019 in Suva (*n* = 5), and 1,880 to 2018 (*n* = 3) in Savusavu.

Coral habitats in Suva did not greatly change over the course of our study. Edge length increased slightly in Suva between 1875 and 2019 (χ^2^, *p* < .001); however, edge density was highly variable in the same time period, with no clear pattern (χ^2^, *p* = .88). Coral area increased by about 8% (+40 ha, χ^2^, *p* < .001) and core area similarly increased (8%, +34.7 ha, χ^2^, *p* < .001). The overall number of patches, and related patch density, varied highly among charts from 1875 to 2019, causing no statistical difference in values (χ^2^, *p* = .72, *p* = .99, respectively).

**FIGURE 4:**
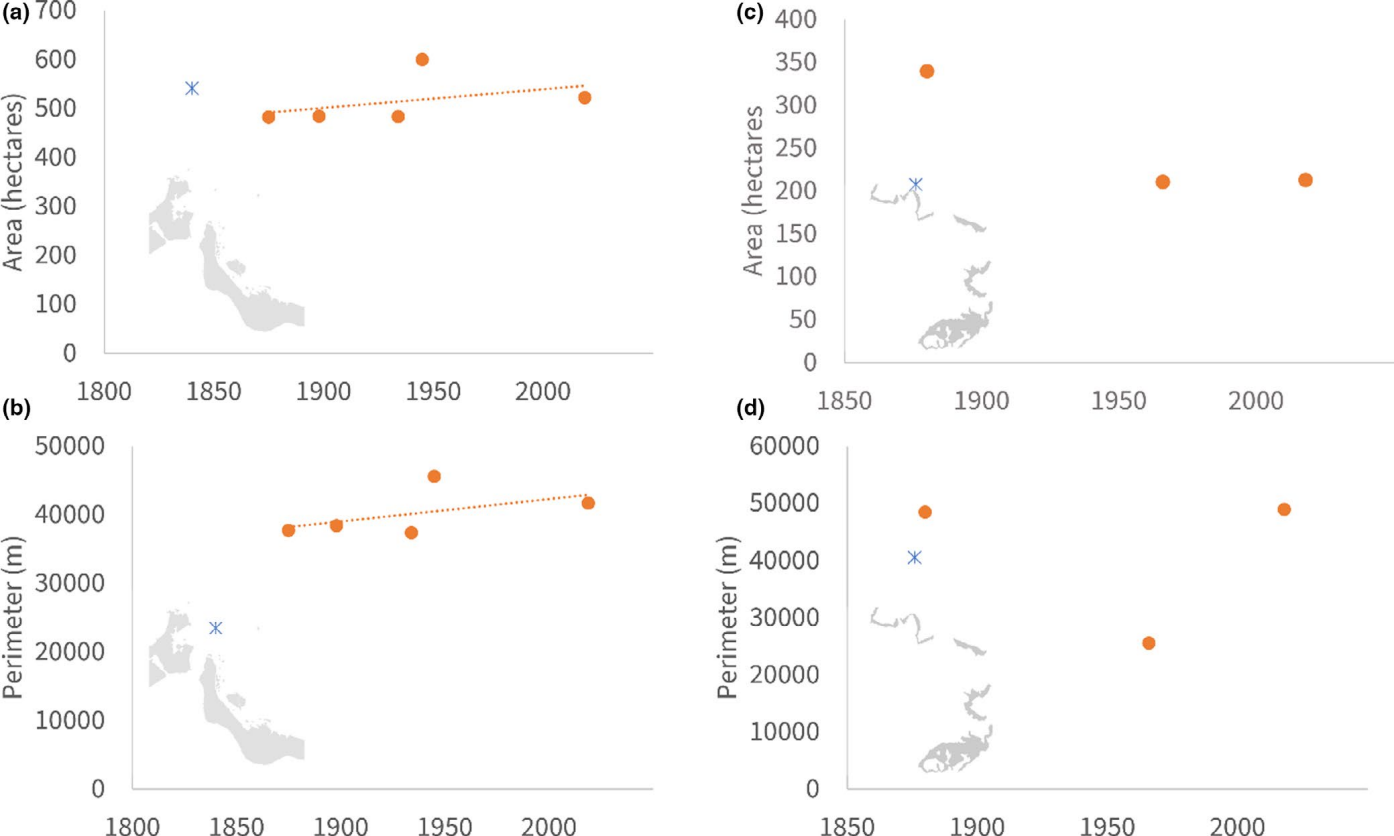
Area (top; a, c) and perimeter (bottom; b, d) of coral reefs in Suva (left; a, b) and Savusavu (right; c, d). Earliest charts, which depicted less detail than the latest charts, are shown as X. Gray backgrounds identify the location but are not to scale

**FIGURE 5:**
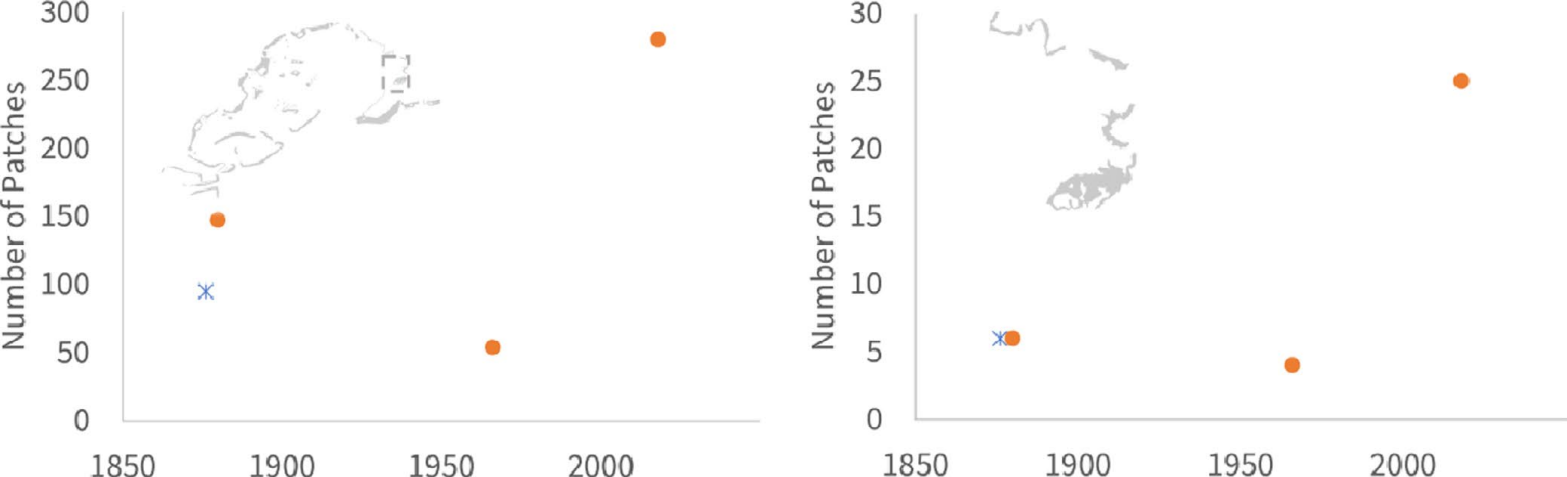
Number of reef habitat patches in Savusavu, in the wider view (left) and nearshore (right). Inset box shows the location of nearshore Savusavu on the wider chart. Earliest charts, which depicted less detail than the latest charts, are shown as X. Gray backgrounds identify the location but are not to scale

In contrast, coral habitats adjacent to the city of Savusavu saw major losses in both nearshore reefs (Figure 4; χ^2^, *p* < .001) and across all of Savusavu Bay, which lost 2,933 hectares of reef area from 1,880 to 2018. Total edge length was variable during this time period (Figure 4), likely as a result of the decrease in coral area combined with a 90% increase in the total number of patches (Figure 5; χ^2^, *p* < .001).

In addition to reef habitat, we examined change in the extent of mangroves and hardened shoreline on the coastlines of Suva and Savusavu. Seven charts from Suva, starting in 1898, and three charts from Savusavu, starting in 1,880, showed mangrove habitats and were used for this analysis (Table 1).

**FIGURE 6:**
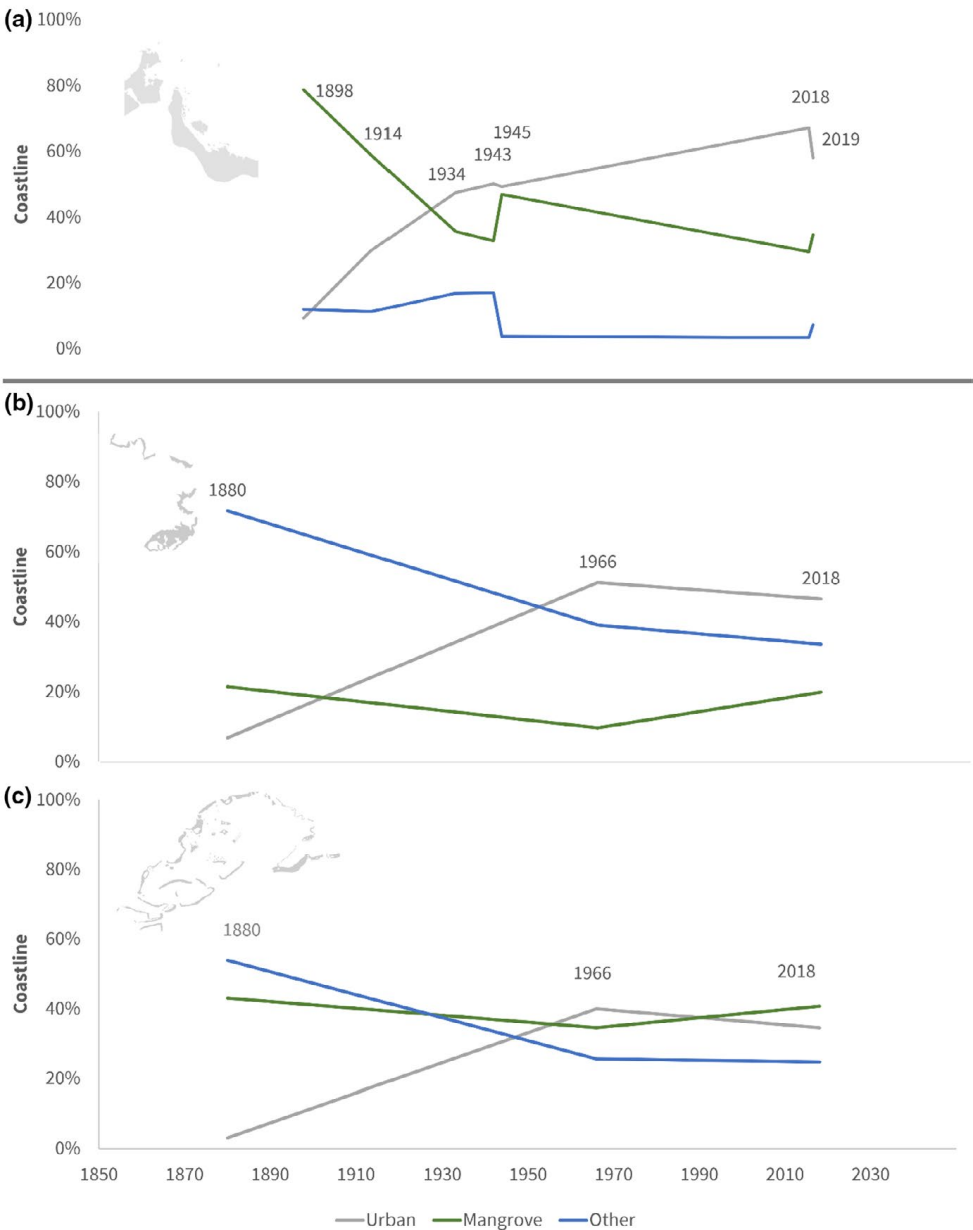
Coastline use in Suva (top; a) and Savusavu (bottom; b, c). In Savusavu, mangroves represented a smaller part of the coastline closer to town (b) than over the entire bay (c). Coastline is occupied by either urban infrastructure, mangrove forests, or other, natural or agricultural landscapes. Gray backgrounds identify the location but are not to scale

In Suva, mangroves represented 79% of the coastline in 1898, but just 35% by 2019 (Figure 6). Simultaneously, urbanized coastline grew from 9% to 58%. Most of this growth occurred in the city center, where seawalls replaced mangroves on the shoreline. Other coast use remained relatively stable during this period, accounting for a maximum of 17% of the coast in 1943. The decline in mangroves did not correlate well with year (lm, −0.299, *p* = .13, adjusted *r* ^2^ = .48), though increase in urban space was correlated slightly better (lm, 0.003, *p* = .056, adjusted *r* ^2^ = .64).

Alternatively, in the city of Savusavu, mangrove habitat remained relatively stable at an average 17% (3.7% *SE*, χ^2^, *p* = .09) of the shoreline near town. Hardened shoreline increased significantly in 1966 from 7% to 51% of the shore, with an accompanied drop in other type of shoreline, from 72% to 39% (χ^2^, *p* < .001; Figure 6). The wider entirety of Savusavu Bay exhibited a similar pattern, with hardened coastlines increasing from 3% to 40% in the 1960s. Farther from the town, mangroves represented a larger proportion of the shore (average 40%, 2.5% SE), but did not drastically change between 1880 and 2018.

## 4 DISCUSSION

Our work shows a pattern of environmental change consistent with an increase in human impact within Suva and Savusavu waters. We see that while coral remained relatively stable in Suva, mangroves once spanning nearly the entire coast were reduced by more than 50%. On the other hand, Savusavu reefs show major losses in area and increased reef fragmentation, and while mangroves were only slightly affected by urbanization on the coastline in Savusavu, both places saw a marked increase in hardened land/sea interface. Taken together these findings suggest that the coastal environments of Suva and Savusavu have become degraded over a century and a half of commercial use. Our results reflect the complex and often idiosyncratic nature of ecological systems and reinforce the need for quantifiable data from historical sources.

Despite reports of the potential degradation of Suva reefs (see Naidu & Morrison, 1994), historical charts of Suva harbor revealed a moderately stable amount of reef area, a potential positive sign that increasing urbanization has not had a major impact on historically available habitat area, regardless of current coral health, sedimentation, or bleaching. Geospatial data like those used in this study cannot reveal coral status or health through time, but no change in area over time reflects that the reefs remain built‐up enough to warrant navigational identification and that the reefs, dead or alive, may still offer ecosystem services like shoreline protection. The stability of coral area in Suva may reflect a difference in dominant coral genus, like *Porites* spp. (Goberdhan & Kininmonth, 2021), whose solid mounding morphology may have been of greater interest to cartographers or be less susceptible to outright destruction, loss, and erosion than tree and plate morphologies common in Savusavu. Recent studies in Suva have shown that coral recruitment has also been little affected by sedimentation and pollution (Lal et al., 2018), suggesting that the reef may be more able to withstand degradation than previously thought. On the other hand, we saw that in Savusavu major losses in coral area occurred on reefs immediately adjacent to the town and on larger scale charts, where reefs are generally considered to be in good condition (see Goberdhan & Kininmonth, 2021). Savusavu was found to be most affected by major warming events in 2000, as 40% of scleractinian corals died as a result of bleaching (Cumming et al., 2006). Paired with evidence of increased fragmentation, these results could signal that major changes in biodiversity related to habitat availability have been occurring in Savusavu for the last 50 to 100 years.

Habitat loss and fragmentation of reefs observed around Savusavu has many potentially negative environmental consequences. Past studies have shown strong relationships between species richness and habitat area (Bellwood & Hughes, 2001; Knowlton, 2001). While habitat loss causes declines in fish abundance, diversity, and coral richness, fragmentation can have mixed impacts on ecosystems (Bonin et al., 2011; Caley et al., 2001). Increased habitat fragmentation has been shown to increase immigration and increase coral recruit survival (Bonin et al., 2011). Edge habitat supports fish species that prefer sandy habitat and can be more complex than interior reef habitat (Ault & Johnson, 1998; Friedlander & Parrish, 1998) although edge habitat has been observed to have lower coral cover and more soft coral (Sambrook et al., 2016). Increased habitat heterogeneity and patchiness may result in increased diversity at fine scales, although the limit of this phenomenon is not known and some of this increased richness may come at the cost of interior‐adapted species like damselfish and other planktivores (Sambrook et al., 2016). Historical estimates of coral habitat loss can be used to assess site vulnerability, identify causes of loss, and develop effective conservation efforts (e.g., Bromberg & Bertness, 2005). Setting a healthy baseline of habitat extent and composition is critical to efficiently protecting and preventing further harm and loss in these systems.

We observed an overwhelming loss of mangrove habitat in Suva, confirming general trends in mangrove habitat losses throughout the Pacific Islands and in Fiji in particular (Lal, 1990). In Suva, our results showed that the loss of mangroves was poorly correlated with year; however, this result is unsurprising since continuous, slow yearly loss is much less likely than periodic forest destruction. Nonetheless, this relationship shows a significant loss in mangrove habitat from 1898 to today. The difference in change in mangrove coastline between Suva and Savusavu reflects the disparate histories of each place. In Suva, the growing capital city directly replaced mangrove habitat on the shoreline, while in Savusavu mangroves were not greatly affected by the small increase in urbanized space due to the many available acres of abandoned farmland. However, our data in Savusavu jump from 1880 to 1962 and may not accurately reflect early losses in mangrove habitat to agriculture that began as early as 1896 (Lal, 1990). Indeed, our data do show a slight decline in mangroves during this period (43% of coastline to 35%) and further exploration into the replacement of mangroves in this area is warranted. Although our research helps to provide historical context for shoreline use through time, our measurements of linear coastline occupation may not adequately capture loss in mangrove forest area. Further research on historical mangrove habitat loss could use aerial photographs to evaluate area and contemporary studies of loss have used Landsat imagery to document mangrove change since 2000 (Cameron et al., 2021).

The replacement of mangrove habitat with urban infrastructure in Suva reflects the decades‐long influence of European colonization. Evidence from historical photographs (Figure 1; Supporting Information) shows that the transformation from mangrove‐lined coasts and lush interiors to sea‐walled shorelines and paved roads began as early as 1914 in Suva. One effect of urbanization in the tropics during the 20th century was the hardening of mangrove coastlines, which led to difficulties managing erosion (e.g., Thampanya et al., 2006) and loss of beneficial ecosystem services to native communities. Mangroves mediate storm surge (Granek & Ruttenberg, 2007), provide habitat for commercially valuable species (Carney, 2017), influence nutrient flows (Feller et al., 1999), serve as carbon sinks (Alongi, 2014), and provide juvenile habitat for fisheries (Mumby, 2006). Mangrove forest destruction is accompanied by economic losses from fisheries yield, higher storm protection costs, and loss of building materials, even when mangroves are replaced by economic ventures like agriculture and aquaculture (see Lal, 1990). Additionally, urban mangroves are less efficient in supporting reef fish recruitment than rural ones (Brooker et al., 2020). Continued development and building plans in Pacific nations should consider building sustainably within mangrove forests, to the benefit of and with input from Indigenous communities and to be better adaptable to rising sea levels.

Hardened shorelines also increase the risk of colonization by non‐native species, which are a growing threat to coral reefs and biodiversity (Goldberg & Wilkinson, 2004). As a major port city, Suva is at a higher risk of invasion than Savusavu due to vectors related to shipping (i.e., ballast and hull fouling); however, both cities saw an increase in hardened coasts. In Fiji, only a few non‐native species have been identified in published literature, including *Mytilopsis sallei* (Bax et al., 2002), *Ostrea edulis* (Bromley et al., 2016), *Gracilaria edulis, Sargassum polycystum* (Charan et al., 2017), and *Kappaphycus* spp. (Sulu et al., 2003) but this could be due to a lack of taxonomic knowledge and surveys of the area. Invasive species pressure in tropical ports is still poorly understood (Hutchings et al., 2002), and as a major Pacific port city, further research in Suva would help clarify the invasion risk to tropical coastal habitats.

Performing an historical ecology analysis brings many challenges. As historical ecologists, our work depends on the largesse and expertise of our predecessors, yet accessing nontraditional data sources can be a useful approach toward marine conservation biology (McClenachan et al., 2015; Thurston et al., 2015). Historical charts offer otherwise unavailable glimpses into the past, but in the elapsed time since creation important context and detail is lost, and commonly map reliability declines farther back in time (Lukas, 2014). Few details exist about the method of cartography used to create charts, or level of uncertainty warranted per data point, though charts created for military and navigational use are likely unbiased (Vellend et al., 2013). Despite being created for similar purposes, each chart likely reflects the biases and interests of each cartographer, confusing temporal comparisons. Editorial decisions on symbology and extent are a likely source of some error. Nautical charts require even further scrutiny, as identification of underwater resources is difficult from the surface and may not distinguish between live or dead coral stands. Moreover, despite best practices to fit charts to current GIS layers, our detailed quantitative results are likely skewed by chart detail and projection issues. However, the remarkable qualitative trends we observed in coral loss, fragmentation, mangrove loss, and urban hardening deserve further research and attention. We expect that the directionality and scale of the trends observed in this study overwhelm any possible error from lack of map detail or scale differences (Lukas, 2014). Our results should be reviewed complementary to contemporary in situ studies but nevertheless represent important findings from otherwise unattainable information. Data on coral location and change could be confirmed with sediment cores and radioisotope dating. Further verification of our results should balance historical ecology with experimental and observational studies possible in the field.

### 4.1 *Me bulabula na Lase, Veidogo kei na Lewe ni vanua ena veisiga ni mataka*: A future for healthy corals, mangroves, and people

Our work shows the dramatic changes that the harbors of Suva and Savusavu have undergone in the past century and a half. Different political and ecological histories have resulted in dissimilar forms of change, but in both cases, the harbors of the early 21st century represent dramatically altered environments. During this time, Fijians have seen tremendous changes to their shores including reductions in coastal forests and changes in the character and structure of coral reefs. Fiji is a leader in conservation in the Pacific with ambitious plans to protect both coastal and terrestrial habitat. Our work here demonstrates the historical context within which those ambitions can be framed. By understanding the past, we gain benchmarks against which future restoration and conservation targets may be set. In addition, we honor and respect the land and the seas of Fiji by giving recognition to the ways they have changed and by mapping out a future for healthy corals, mangroves, and human communities.

## CONFLICT OF INTEREST

The authors declare no conflicts of interest.

## AUTHOR CONTRIBUTION

**Katherine N. Lawson:** Conceptualization (equal); Data curation (lead); Formal analysis (lead); Investigation (equal); Methodology (equal); Project administration (equal); Software (equal); Validation (equal); Visualization (equal); Writing‐original draft (equal); Writing‐review & editing (equal). **Haleigh Letendre:** Data curation (supporting); Formal analysis (equal); Methodology (supporting); Software (supporting); Writing‐original draft (supporting). **Joshua A. Drew:** Conceptualization (equal); Data curation (equal); Funding acquisition (lead); Methodology (equal); Project administration (equal); Resources (equal); Supervision (lead); Writing‐original draft (equal); Writing‐review & editing (equal).

## Supporting information

Figure S1‐S6

Appendix S1

Supporting information

## Data Availability

Data can be accessed at https://github.com/newcomerk/HistoricalMaps_Lawsonetal2021. Permanent storage of data used in this paper will also be held by SUNY‐ESF Esploro Research Repository. Information on the charts included in this analysis is available in the Supporting Material.

## References

[ece38153-bib-0001] Alongi, D. M. (2014). Carbon cycling and storage in mangrove forests. Annual Review of Marine Science, 6(1), 195–219. 10.1146/annurev-marine-010213-135020 24405426

[ece38153-bib-0002] Ault, T. R. , & Johnson, C. R. (1998). Spatially and temporally predictable fish communities on coral reefs. Ecological Monographs, 68(1), 25–50. 10.1890/0012-9615(1998)068[0025:SATPFO]2.0.CO;2

[ece38153-bib-0003] Bao, K. , & Drew, J. (2017). Traditional ecological knowledge, shifting baselines, and conservation of Fijian molluscs. Pacific Conservation Biology, 23(1), 81. 10.1071/PC16016

[ece38153-bib-0004] Barrett, J. W. (1935). To Melbourne and back. British Medical Journal, 1(3882), 1147. 10.1136/bmj.1.3882.1147-b

[ece38153-bib-0005] Bax, N. J. , Hayes, K. R. , Marshall, A. , Parry, D. , & Thresher, R. (2002). Man‐made marinas as sheltered islands for alien marine organisms: Establishment and eradication of an alien invasive marine species. In C. R. Veitch & M. N. Clout (Eds.), Turning the tide: The eradication of invasive species: Proceedings of the International Conference on Eradication of Island Invasives (pp. 26–39). IUCN. http://hdl.handle.net/102.100.100/199081?index=1

[ece38153-bib-0006] Bellwood, D. R. , & Hughes, T. P. (2001). Regional‐scale assembly rules and biodiversity of coral reefs. Science, 292(5521), 1532–1535. 10.1126/science.1058635 11375488

[ece38153-bib-0007] Bligh, W. (2013). A Voyage to the South Sea, for the purpose of conveying the bread‐fruit tree to the West Indies. In His Majesty’s ship the Bounty, commanded by Lieutenant William Bligh (Cambridge Library Collection ‐ Maritime Exploration). Cambridge University Press. 10.1017/CBO9781139565578

[ece38153-bib-0008] Bonin, M. C. , Almany, G. R. , & Jones, G. P. (2011). Contrasting effects of habitat loss and fragmentation on coral‐associated reef fishes. Ecology, 92(7), 1503–1512. 10.1890/10-0627.1 21870624

[ece38153-bib-0009] Bromberg, K. D. , & Bertness, M. D. (2005). Reconstructing New England salt marsh losses using historical maps. Estuaries, 28(6), 823–832. 10.1007/BF02696012

[ece38153-bib-0010] Bromley, C. , McGonigle, C. , Ashton, E. C. , & Roberts, D. (2016). Bad moves: Pros and cons of moving oysters – A case study of global translocations of *Ostrea edulis* Linnaeus, 1758 (Mollusca: Bivalvia). Ocean & Coastal Management, 122, 103–115. 10.1016/j.ocecoaman.2015.12.012

[ece38153-bib-0011] Brooker, R. M. , Seyfferth, A. L. , Hunter, A. , Sneed, J. M. , Dixson, D. L. , & Hay, M. E. (2020). Human proximity suppresses fish recruitment by altering mangrove‐associated odour cues. Scientific Reports, 10(1), 21091. 10.1038/s41598-020-77722-7 33273575 PMC7713406

[ece38153-bib-0012] Caley, M. J. , Buckley, K. A. , & Jones, G. P. (2001). Separating ecological effects of habitat fragmentation, degradation, and loss on coral commensals. Ecology, 82(12), 3435–3448. 10.1890/0012-9658(2001)082[3435:SEEOHF]2.0.CO;2

[ece38153-bib-0013] Cameron, C. , Maharaj, A. , Kennedy, B. , Tuiwawa, S. , Goldwater, N. , Soapi, K. , & Lovelock, C. E. (2021). Landcover change in mangroves of Fiji: Implications for climate change mitigation and adaptation in the Pacific. Environmental Challenges, 2, 100018. 10.1016/j.envc.2020.100018

[ece38153-bib-0014] Carney, J. (2017). “The mangrove preserves life”: Habitat of African survival in the Atlantic world. Geographical Review, 107(3), 433–451. 10.1111/j.1931-0846.2016.12205.x

[ece38153-bib-0015] Charan, H. , N’Yeurt, A. D. R. , Iese, V. , & Chopin, T. (2017). The effect of temperature on the growth of two pest seaweeds in Fiji. In 2nd International Conference on Energy, Environment and Climate. 7.

[ece38153-bib-0016] Cheal, A. J. , MacNeil, M. A. , Emslie, M. J. , & Sweatman, H. (2017). The threat to coral reefs from more intense cyclones under climate change. Global Change Biology, 23(4), 1511–1524. 10.1111/gcb.13593 28139035

[ece38153-bib-0017] Cochrane, E. (2017). Ancient Fiji: Melting pot of the Southwest Pacific. Oxford University Press.

[ece38153-bib-0018] Costa, M. , Le Baron, N. , Tenhunen, K. , Nephin, J. , Willis, P. , Mortimor, J. P. , Dudas, S. , & Rubidge, E. (2020). Historical distribution of kelp forests on the coast of British Columbia: 1858–1956. Applied Geography, 120, 102230. 10.1016/j.apgeog.2020.102230

[ece38153-bib-0019] Cumming, R. L. , Toscano, M. A. , Lovell, E. R. , Carlson, B. A. , Dulvy, N. K. , Hughes, A. , Koven, J. F. , Sykes, H. R. , Taylor, O. J. S. , & Vaughan, D. (2006). Mass coral bleaching in the Fiji Islands, 2000: 8.

[ece38153-bib-0020] Dacks, R. , Ticktin, T. , Jupiter, S. D. , & Friedlander, A. M. (2020). Investigating the role of fish and fishing in sharing networks to build resilience in coral reef social‐ecological systems. Coastal Management, 48(3), 165–187. 10.1080/08920753.2020.1747911

[ece38153-bib-0021] Dana, J. D. (1849). United States Exploring Expedition: During the years 1838, 1839, 1840, 1841, 1842 under the command of Charles Wilkes. Atlas zoophytes. C. Sherman. PMC10420750 38209897

[ece38153-bib-0022] Davis, M. T. , Newell, P. F. , & Quinn, N. J. (1999). TBT contamination of an artisanal subsistence fishery in Suva harbour, Fiji. Ocean & Coastal Management, 42(6), 591–601. 10.1016/S0964-5691(99)00035-6

[ece38153-bib-0023] Davis, W. M. (1918). The reef‐encircled islands of the Pacific. Journal of Geography, 17(2), 58–68. 10.1080/00221341808984389

[ece38153-bib-0024] Duke, N. C. , Meynecke, J.‐O. , Dittmann, S. , Ellison, A. M. , Anger, K. , Berger, U. , Cannicci, S. , Diele, K. , Ewel, K. C. , Field, C. D. , Koedam, N. , Lee, S. Y. , Marchand, C. , Nordhaus, I. , & Dahdouh‐Guebas, F. (2007). A world without mangroves? Science, 317(5834), 41b–42b. 10.1126/science.317.5834.41b 17615322

[ece38153-bib-0025] Feller, I. C. , Whigham, D. F. , O’Neill, J. P. , & McKee, K. L. (1999). Effects of nutrient enrichment on within‐stand cycling in a mangrove forest. Ecology, 80(7), 2193–2205. 10.1890/0012-9658(1999)080[2193:EONEOW]2.0.CO;2

[ece38153-bib-0026] Friedlander, A. , & Parrish, J. (1998). Habitat characteristics affecting fish assemblages on a Hawaiian coral reef. Journal of Experimental Marine Biology and Ecology, 224, 1–30. 10.1016/S0022-0981(97)00164-0

[ece38153-bib-0027] Goberdhan, L. , & Kininmonth, S. (2021). Insights into coral growth rate trends in Fiji. Coral Reefs, 40(1), 251–266. 10.1007/s00338-020-02037-y

[ece38153-bib-0028] Goldberg, J. , & Wilkinson, C. (2004). 1 Global threats to coral reefs: coral bleaching, global climate change, disease, predator plagues, and invasive species. Status of Coral Reefs of the World: 2004: 26.

[ece38153-bib-0029] Graci, S. , & Vliet, L. V. (2020). Examining stakeholder perceptions towards sustainable tourism in an island destination. The Case of Savusavu, Fiji. Tourism Planning & Development, 17(1), 62–81. 10.1080/21568316.2019.1657933

[ece38153-bib-0030] Granek, E. F. , & Ruttenberg, B. I. (2007). Protective capacity of mangroves during tropical storms: A case study from ‘Wilma’ and ‘Gamma’ in Belize. Marine Ecology Progress Series, 343, 101–105. 10.3354/meps07141

[ece38153-bib-0031] Hesselbarth, M. , Sciaini, M. , Wiengand, K. , & Nowosad, J. (2019). landscapemetrics: An open‐source R tool to calculate landscape metrics. Ecography, 42, 1648–1657 (ver. 0).

[ece38153-bib-0032] Hutchings, P. A. , Hilliard, R. W. , & Coles, S. L. (2002). Species introductions and potential for marine pest invasions into tropical marine communities, with special reference to the Indo‐Pacific. Pacific Science, 56(2), 223–233. 10.1353/psc.2002.0017

[ece38153-bib-0033] Inskip, T. W. H. (1937). Strategic importance of Pacific Islands report by New Zealand Government. In Institute of Latin American Studies. Retrieved from https://sas‐space.sas.ac.uk/8288/. Accessed 10 Feb 2021

[ece38153-bib-0034] Jenny, B. , & Hurni, L. (2011). Studying cartographic heritage: Analysis and visualization of geometric distortions. Computers & Graphics, 35(2), 402–411. 10.1016/j.cag.2011.01.005

[ece38153-bib-0035] Klein, E. S. , & Thurstan, R. H. (2016). Acknowledging long‐term ecological change: The problem of shifting baselines. In K. Schwerdtner Máñez & B. Poulsen (Eds.), Perspectives on oceans past (pp. 11–29). Springer. 10.1007/978-94-017-7496-3_2

[ece38153-bib-0036] Knowlton, N. (2001). Coral reef biodiversity–Habitat size matters. Science, 292(5521), 1493–1495. 10.1126/science.1061690 11379628

[ece38153-bib-0037] Lal, P. N. (1990). Conservation or conversion of mangroves in Fiji: An ecological economic analysis. Environment and Policy Institute, East‐West Center.

[ece38153-bib-0038] Lal, R. , Kininmonth, S. , N’Yeurt, A. D. R. , Riley, R. H. , & Rico, C. (2018). The effects of a stressed inshore urban reef on coral recruitment in Suva Harbour, Fiji. Ecology and Evolution, 8(23), 11842–11856. 10.1002/ece3.4641 30598781 PMC6303754

[ece38153-bib-0039] Lukas, M. C. (2014). Cartographic reconstruction of historical environmental change. Cartographic Perspectives, 78, 5–24. 10.14714/CP78.1218

[ece38153-bib-0040] McClenachan, L. , Cooper, A. B. , McKenzie, M. G. , & Drew, J. A. (2015). The importance of surprising results and best practices in Historical Ecology. BioScience, 65(9), 932–939. 10.1093/biosci/biv100

[ece38153-bib-0041] McClenachan, L. , O’Connor, G. , Neal, B. P. , Pandolfi, J. M. , & Jackson, J. B. C. (2017). Ghost reefs: Nautical charts document large spatial scale of coral reef loss over 240 years. Science Advances, 3(9), e1603155. 10.1126/sciadv.1603155 28913420 PMC5587093

[ece38153-bib-0042] Mumby, P. J. (2006). Connectivity of reef fish between mangroves and coral reefs: Algorithms for the design of marine reserves at seascape scales. Biological Conservation, 128(2), 215–222. 10.1016/j.biocon.2005.09.042

[ece38153-bib-0043] Munday, P. L. (2004). Habitat loss, resource specialization, and extinction on coral reefs. Global Change Biology, 10(10), 1642–1647. 10.1111/j.1365-2486.2004.00839.x

[ece38153-bib-0044] Naidu, S. D. , & Morrison, R. J. (1994). Contamination of Suva harbour, Fiji. Marine Pollution Bulletin, 29(1), 126–130. 10.1016/0025-326X(94)90436-7

[ece38153-bib-0045] O’Garra, T. (2009). Bequest values for marine resources: How important for Indigenous communities in less‐developed economies? Environmental & Resource Economics, 44, 179–202. 10.1007/s10640-009-9279-3

[ece38153-bib-0046] Palandro, D. A. , Andréfouët, S. , Hu, C. , Hallock, P. , Müller‐Karger, F. E. , Dustan, P. , Callahan, M. K. , Kranenburg, C. , & Beaver, C. R. (2008). Quantification of two decades of shallow‐water coral reef habitat decline in the Florida Keys National Marine Sanctuary using Landsat data (1984–2002). Remote Sensing of Environment, 112(8), 3388–3399. 10.1016/j.rse.2008.02.015

[ece38153-bib-0047] Pauly, D. (1995). Anecdotes and the shifting baseline syndrome of fisheries. Trends in Ecology & Evolution, 10(10), 430. 10.1016/S0169-5347(00)89171-5 21237093

[ece38153-bib-0048] Penn, N. (1983). The environmental consequences and management of coral sand dredging in the Suva region. Diss University College of Swansea. 308.

[ece38153-bib-0049] Prasad, R. D. , & Raturi, A. (2019). Low carbon alternatives and their implications for Fiji’s electricity sector. Utilities Policy, 56, 1–19. 10.1016/j.jup.2018.10.007

[ece38153-bib-0050] Pratchett, M. S. , Hoey, A. S. , Wilson, S. K. , Messmer, V. , & Graham, N. A. J. (2011). Changes in biodiversity and functioning of reef fish assemblages following coral bleaching and coral loss. Diversity, 3(3), 424–452. 10.3390/d3030424

[ece38153-bib-0051] R Core Team (2020). R: A language and environment for statistical computing. R Foundation for Statistical Computing. Retrieved from https://www.R‐project.org/

[ece38153-bib-0052] Rhodes, K. L. , Hernandez‐Ortiz, D. X. , Cuetos‐Bueno, J. , Ioanis, M. , Washington, W. , & Ladore, R. (2018). A 10‐year comparison of the Pohnpei, Micronesia, commercial inshore fishery reveals an increasingly unsustainable fishery. Fisheries Research, 204, 156–164. 10.1016/j.fishres.2018.02.017

[ece38153-bib-0053] Sambrook, K. , Jones, G. P. , & Bonin, M. C. (2016). Life on the edge: Coral reef fishes exhibit strong responses to a habitat boundary. Marine Ecology Progress Series, 561, 203–215. 10.3354/meps11940

[ece38153-bib-0054] Sangha, K. K. , Maynard, S. , Pearson, J. , Dobriyal, P. , Badola, R. , & Hussain, S. A. (2019). Recognising the role of local and Indigenous communities in managing natural resources for the greater public benefit: Case studies from Asia and Oceania region. Ecosystem Services, 39, 100991. 10.1016/j.ecoser.2019.100991

[ece38153-bib-0055] Seemann, B. (1862). Viti: An account of a government mission to the Vitian or Fijian Islands, in the years 1860‐61. Macmillan & Co.

[ece38153-bib-0100] Shuler, C. K. , Amato, D. W. , Gibson, V. , Baker, L. , Olguin, A. N. , Dulai, H. , Smith, C. M. , & Alegado, R. A. (2019). Assessment of terrigenous nutrient loading to coastal ecosystems along a human land‐use gradient, Tutuila, American Samoa. Hydrology, 6(1), 18. 10.3390/hydrology6010018

[ece38153-bib-0056] Smith, J. W. (2013). The bound[less] sea: Wilderness and the United States exploring expedition in the Fiji Islands. Environmental History, 18(4), 710–737. 10.1093/envhis/emt067

[ece38153-bib-0057] Spalding, M. D. , & Brown, B. E. (2015). Warm‐water coral reefs and climate change. Science, 350(6262), 769–771. 10.1126/science.aad0349 26564846

[ece38153-bib-0058] Sparling, C. H. (1923). A short cruise in the South Seas. The Geographical Teacher, 12(1), 60–64.

[ece38153-bib-0059] Sulu, R. , Kumar, L. , Hay, C. , & Pickering, T. (2003). Kappaphycus seaweed in the Pacific: review of introductions and field testing proposed quarantine protocols. Noumea: Secretariat of the Pacific Community:85.

[ece38153-bib-0060] Thampanya, U. , Vermaat, J. E. , Sinsakul, S. , & Panapitukkul, N. (2006). Coastal erosion and mangrove progradation of Southern Thailand. Estuarine, Coastal and Shelf Science, 68(1), 75–85. 10.1016/j.ecss.2006.01.011

[ece38153-bib-0061] Thurstan, R. H. , McClenachan, L. , Crowder, L. B. , Drew, J. A. , Kittinger, J. N. , Levin, P. S. , Roberts, C. M. , & Pandolfi, J. M. (2015). Filling historical data gaps to foster solutions in marine conservation. Ocean & Coastal Management, 115, 31–40. 10.1016/j.ocecoaman.2015.04.019

[ece38153-bib-0062] Vellend, M. , Brown, C. D. , Kharouba, H. M. , McCune, J. L. , & Myers‐Smith, I. H. (2013). Historical ecology: Using unconventional data sources to test for effects of global environmental change. American Journal of Botany, 100(7), 1294–1305. 10.3732/ajb.1200503 23804553

[ece38153-bib-0063] Ward, R. G. (1959). The population of Fiji. Geographical Review, 49(3), 322–341. 10.2307/211910

[ece38153-bib-0064] Whipple, A. A. , Grossinger, R. M. , & Davis, F. W. (2011). Shifting baselines in a California Oak Savanna: Nineteenth century data to inform restoration scenarios. Restoration Ecology, 19(101), 88–101. 10.1111/j.1526-100X.2009.00633.x

[ece38153-bib-0065] Wilkes, C. (1845). Narrative of the United States’ Exploring Expedition: During the years 1838, 1839, 1840, 1841, 1842. Whittaker. PMC10420750 38209897

