# Supplementary material for: Historical Maps provide insight into a century and a half of habitat change in Fijian coasts
Source: Ecol Evol. 2021 Oct 29;11(22):15573–84. doi: 10.1002/ece3.8153 (PMC8601896; doi:10.1002/ece3.8153)

Supporting Fig. 1. Coral area in Suva through all time points in this study (a: 1840, not statistically analyzed; b: 1875; c: 1898; d: 1934; e: 1945; f: 2019). Background is a current grayscale basemap.

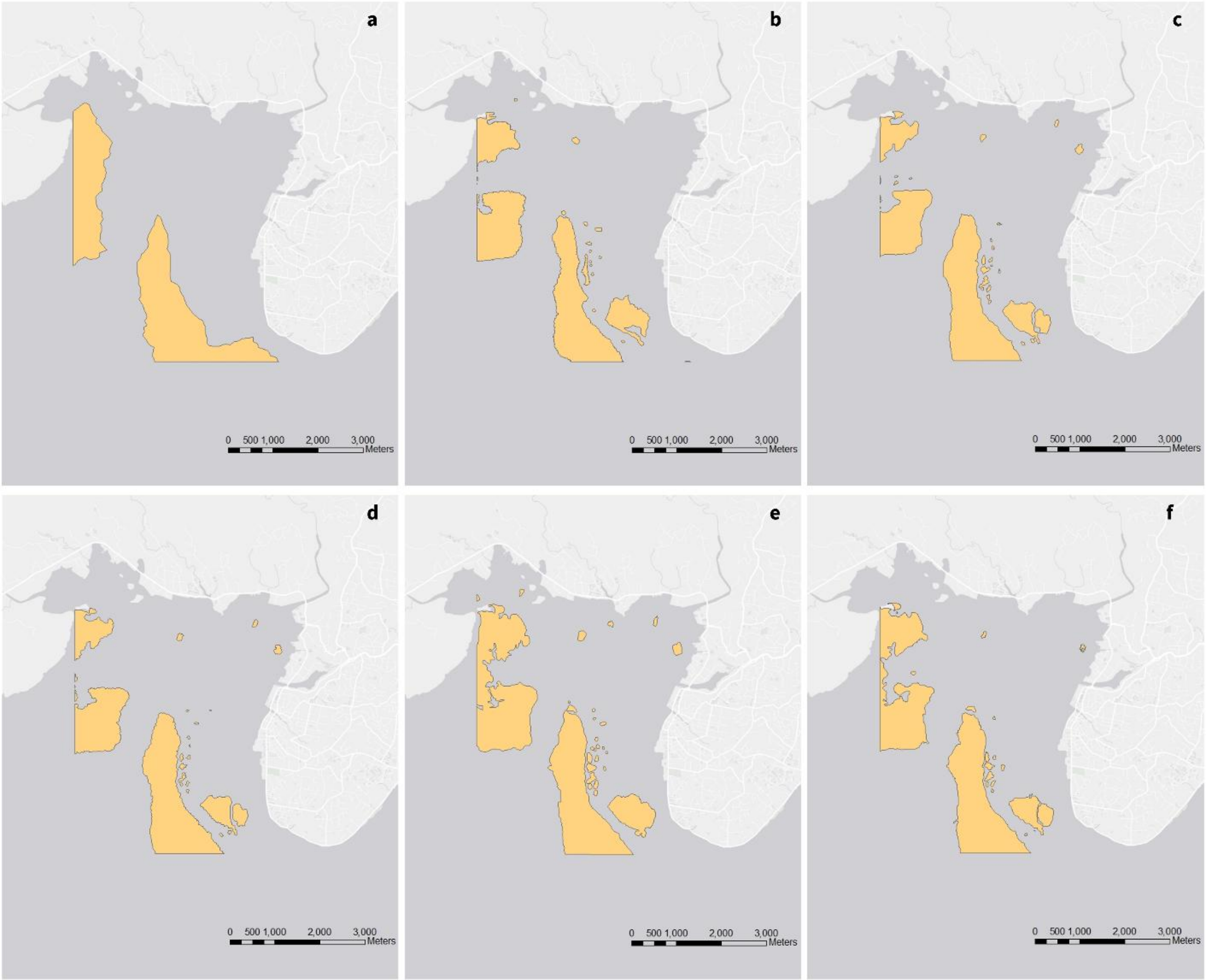

Supporting Fig. 2. Coral area in Savusavu nearshore through all time points analyzed in this study (a: 1876, not statistically analyzed; b: 1880; c: 1966; d: 2018). Background is a current grayscale basemap.

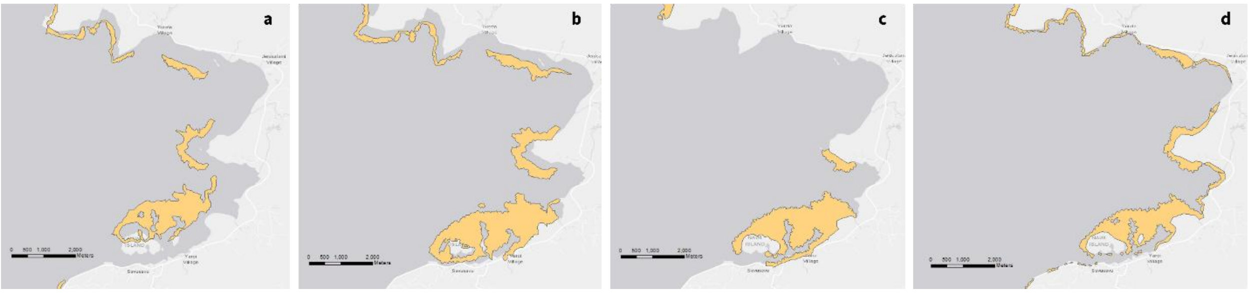

Supporting Fig. 3. Coral area in Savusavu Bay through all time points analyzed in this study (a: 1876, not statistically analyzed; b: 1880; c: 1966; d: 2018). Background is a current grayscale basemap.

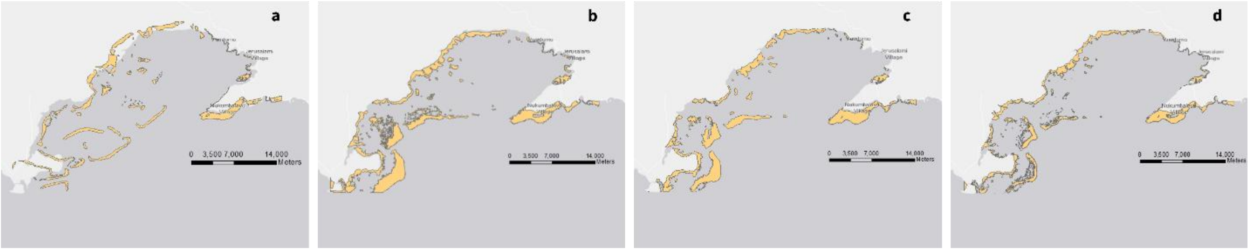

Supporting Fig. 4. Coastline use in Suva through all time points analyzed in this study (a: 1898 b: 1914; c: 1934; d: 1943; e: 1945; g: 2019). Mangroves are green, Urban areas grey, and Other blue. Background is a current grayscale basemap.

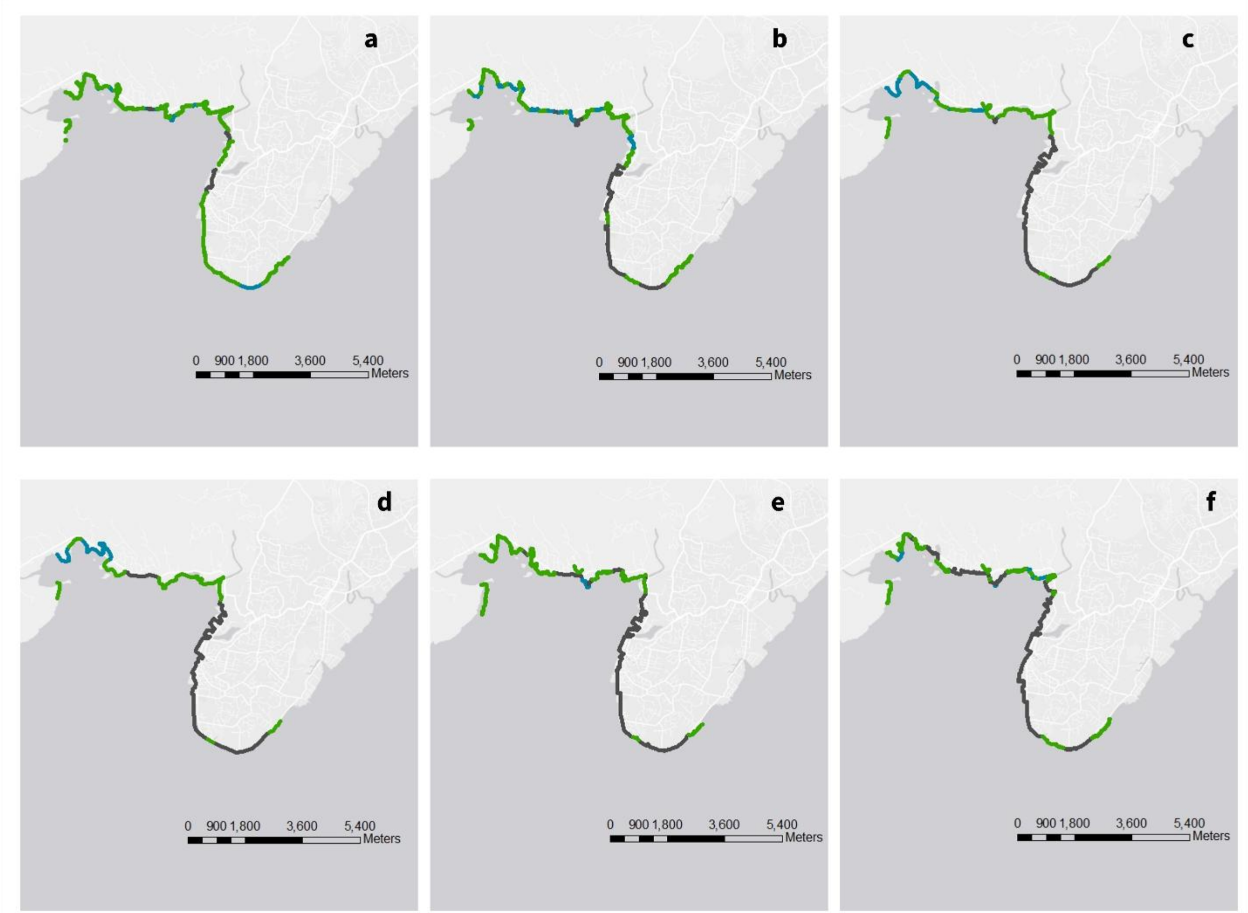

Supporting Fig. 5. Coastline use in Savusavu nearshore through all time points analyzed in this study (a: 1880; b: 1966; c: 2018). Mangroves are green, Urban areas grey, and Other blue. Background is a current grayscale basemap.

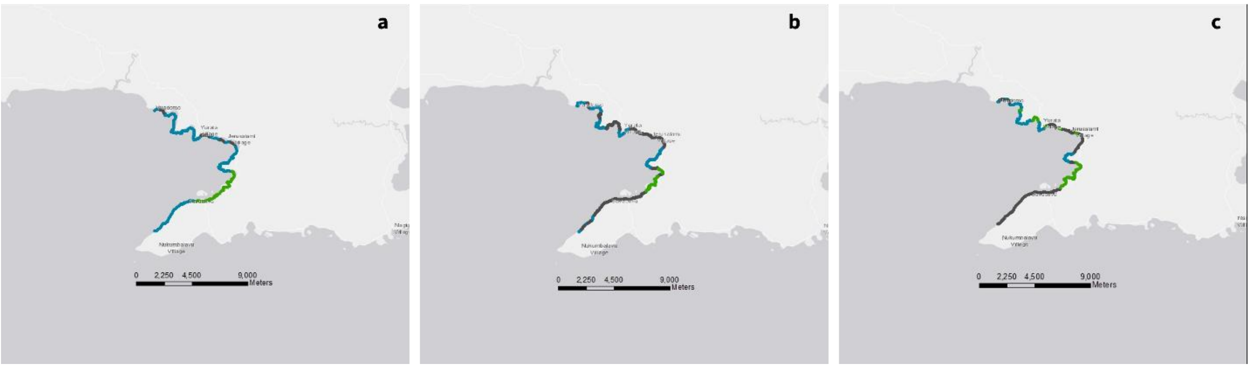

Supporting Fig. 6. Coastline use in Savusavu Bay through all time points analyzed in this study (a: 1880; b: 1966; c: 2018). Mangroves are green, Urban areas grey, and Other blue. Background is a current grayscale basemap.

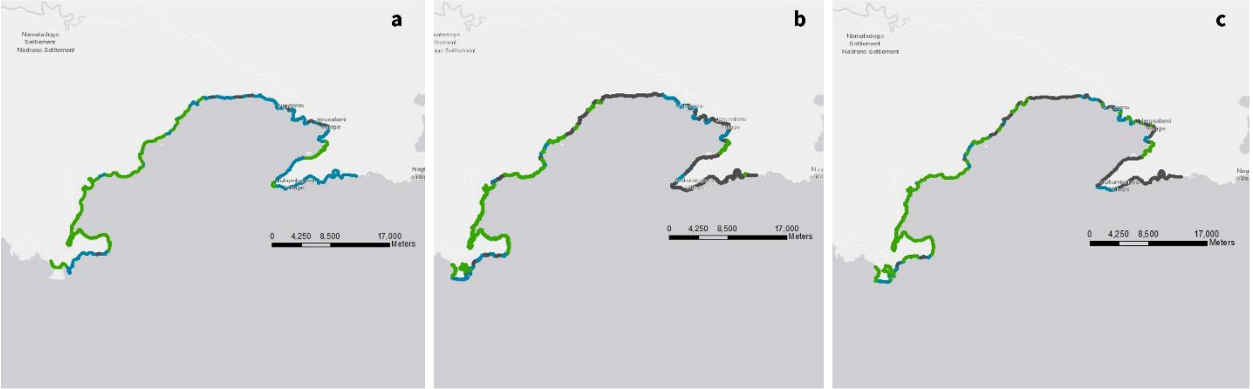

Supplement: Supplementary file 1 — Figure S1‐S6 [file ECE3-11-15573-s003.pdf]
