# Supplementary material for: Historical Maps provide insight into a century and a half of habitat change in Fijian coasts
Source: Ecol Evol. 2021 Oct 29;11(22):15573–84. doi: 10.1002/ece3.8153 (PMC8601896; doi:10.1002/ece3.8153)
Supplement: Supplementary file 2 — Appendix S1 [file ECE3-11-15573-s001.pdf]

Appendix S1

Publication Year	Location	Object	Copyright	Title	Details	Source	Link
1921	Savusavu	Map	Public Domain	Fiji Islands: Vanua Levu: Savusavu Bay	3rd edition of Map No. 2853 From a British Survey in 1880, United States Hydrographic Office	UC San Diego Library Digital Collection	https://library.ucsd.edu/dc/object/bb87043304
1890	Suva	Photo	Out of Copyright	View in Suva, Fiji approximately 1890 by Charles Kerry	Kerry & Co Photo 308 Geo St. Sydney. Bib ID 7248757	National Library of Australia	https://catalogue.nla.gov.au/Record/7248757
1914	Suva	Photo	Public Domain	Town of Suva and ships	Accession Number P02613.002	Australian War Museum	https://www.awm.gov.au/collection/C347500
1956	Suva	Photo	CC BY-NC, https://creativecommons.org/licens	1956-08-25. SUVA, FIJI. AERIAL BOW VIEW OF THE AIRCRAFT CARRIER HMAS	Cropped from original, Accession Number 301442	Australian War Museum	https://www.awm.gov.au/collection/C232984

			es/by-nc/3.0/au/legalcode	SYDNEY (III)			
1888	Savusavu	Postcard	Out of Copyright	Picturesque Fiji	Melbourne: Algred Martin Ebsworth, Artwork, Print published in the Australasian sketcher, Accession no: A/S27/12/88/204	State Library of Victoria	https://viewer.slv.vic.gov.au/?entity=IE786850&mode=browse
1966	Savusavu	Photo	In copyright, Approved for use in this publication	Fiji Airways plane on landing strip, Savusavu, Fiji, 1966 by Michael Terry	From a collection of expeditions and travels of Michael Terry Bib ID 6980815	National Library of Australia	https://catalogue.nla.gov.au/Record/6980815
