# Supplementary material for: Historical Maps provide insight into a century and a half of habitat change in Fijian coasts
Source: Ecol Evol. 2021 Oct 29;11(22):15573–84. doi: 10.1002/ece3.8153 (PMC8601896; doi:10.1002/ece3.8153)
Supplement: Supplementary file 3 — Supporting information [file ECE3-11-15573-s002.docx]

**3Historical Maps provide insight into a century and a half of habitat change in Fijian coasts**

Katherine N. Lawson^1, 2^, Haleigh Letendre^1^, Joshua A. Drew^1^

^1^ 1 Forestry Dr., Department of Environmental and Forest Biology, State University of New York College of Environmental Science and Forestry, Syracuse, NY 13210

^2^Author for correspondence kanewcom@syr.edu

Orcid ID: KN Lawson 0000-000304062, JA Drew 0000-0001-9072-0885

**Running Head:** Historical Maps of Fiji

**Na uto ni tukutuku**

1. Na veinaki se tatadra eso ni maroroi me vakavotukana se vakaibalebale, ena gadreva na kena veidutaitaki na veivakadidike eso me baleta nai yaubula mai na dua na gauna balavu, kei na kedrai tukutuku duidui mai na veiyasai vuravura eso. Na macala ni veivakadidike eso kei nai tukutuku eso e vakasokumuni ena gauna sa oti yani, ena vukea na kena vakaqaqacotaki ka vakavotukanataki na vakanananu ni kena maroroi se taqomaki nai yaubula duidui eso.
2. Ni railesuvi na kena vakayagataki se vakatorocaketaki na qele ena veigauna sa oti kei na kena veimatasawa e Suva kei Savusavu, e ra veidutaitaki na veitukutuku makawa eso e vakasokumuni taumada, oqo me vaka na veiveisau ni veivanua lase eso, na veidogo, kei na kena vakarabailevutaki se vakatorocaketaki na veibaravi. E vakayagataki edau nai walewale se yaragi ni veiqaravi ka vakatokai na Geographic Information Systems (GIS) me vakatauvataki na veika lelevu eso e sotavi mai na yabaki 1840 I Suva kei na na yabaki 1876 mai Savusavu.
3. E dina ni vakatakila na macala ni vakadidike, na tubu cake ni bula ena koro turaga e Suva, ia na veicakau e sega ni veisau ena loma ni 150 na yabaki, ia na revurevu ni veivakatorocaketaki e kovuta ni rauta e 50% ni veidogo. E laurai ni levu sara na kena vakacacani na veicakau mai na tauni i Savusavu ni vakatauvatani kei Suva. E laurai ni sega ni vu mai na veika oqo ena kena vakacacani na veidogo.
4. Na mape era vakarautaki ena vuku ni veimataqali tukutuku era solia, e laurai ni gauna e vakayacori kina na veitalanoa o ya, ka yacova mai nikua, e se sega ga ni kunei se cava e vakavuna na kena yali se veisau nai tuvaki ni veimatasawa e Viti. Na noda veimaliwai na tamata kei na veikabula eda bula veitikivi ena dua na gauna balavu ena vukei ira na daunivakadidike ena kena valuti na veika e vakatokai na Shifting Baseline Syndrome me rawa ni vakadeitaki nai walewale ni se i naki ni kena maroroi se vakayagataki vakamatau na nodai yaubula eso.

**Keywords:** Historical Ecology, coral reef, mangrove, GIS, coastal development, navigational charts

**Vakamacala Taumada**

Na kena levu na veiririko duidui eso, era sotava tiko na veicakau kei na veidogo, e sega ni okati talega na draki veisau (Spalding & Brown 2015), na levu kei na veitaravi ni cagilaba eso kei na kena kaukauwa (Cheal et al. 2017), nai walewale ni qoli, e sega soti ni matau (Rhodes et al. 2018), kei nai walewale ni vakayagataki qele e sega ni matau (Shuler et al. 2017). Na kena maroroi nai yaubula duidui eso ena veicakau kei na veidogo, e dua na usutu bibi ni kena navuci na nodra maroroi ka taqomaki nai yaubula (Knowlton 2001; Munday 2004; Pratchett et al. 2011 Duke et al. 2007). E dua na bolebole levu, na totolo ni veiveisau era sotava tiko nai yaubula oqo, ka okati talega kina na totolo ni kena veisai e sotavi kei na ciqomi ni duidui ni vakadidike taumada (Klein & Thurston 2016). Ena vakananu ni “Shifting Baseline Syndrome” (Pauly 1995), nai taba tamata e ra donuya era na vakamacalataka na kenai tuvaki ena gauna era sotava taumada kina. Na kena sega ni vakaivolataki na veitukutuku se veiveisau eso e sotavi enai matai ni gauna e sega ni vakadeitaki ka ni sega na kenai vakadinadina. Na lailai se kena sega ni vakaivolataki na veiveisau eso era lako curuma na veiyaubula ena dua na gauna sa oti ena vakatokai me tawa rairai ena gauna oqo ni sega na kena vakadinadina. Nai walewale oqo ena vakavuna na kena yali nai tukutuku bibi ni veiveisau eso era lako curumi, ena veitaba tamata duidui eso. E bolea vakalevu na bula ni mamaroroi kei na kena vakayagataki vakamatau nai yaubula, na kena yali se sega ni vakaivolataki se wasei vakaitalanoa se vosa na veitukutuku se vakasama makawa, eso me baleta na veiveisau ni nodrai vakarau ni bula na veiyaubula eso. E dua na usutu levu ni nodra veiqaravi na kena dau vakavuku ena tabana qo na kena vakasokumuni na veitukutuku eso kei na veiveisau eso e lako curumi, me yavutaki kina na veivakanananu ni mamaroroi oqo. E na Pasivika e bolebole levu na lailai na veitukutuku eso ka vakumuni mai na dua na gauna balavu. E bibi na kena vakasokumuni na veitukutuku vaka oqo ni rawa ni valuta na vakanananu ni *Shifting Baseline Syndrome* baleta ni lako sivia mai na veigauna sa oti, ena vuku ni wiliwili ni lewenivanua, kei na veitukutuku eso ka yaco ena dua na gauna balavu sa oti, ka sega ni lewe levu era nanuma. Ena rawa ni yali vakadua e vuqa nai tukutuku makawa eso me baleta na duidui ni bula era lako curuma nai yaubula duidui eso, ke sega ni vakaivolataki nai tukutuku eso e vakasokumuni mai.

Edua nai walewale ni vakasokumuni tukutuku tudei e vakatokai me *geospatial data* ka tauri mai nai taba mai na maliwa lala (Palandro et al. 2008; Whipple et al. 2011). Na veitukutuku vakasokumuni vaka oqo e rawa ni okati talega ena *Geographic Information Systems (GIS)* me maroroi tiko kina. Nai taba mai na maliwa lala e tavoci na 1972, ya na vuna e levu vei ira nai taba ni maliwa lala mai liu e lesu ga na 60 – 70 na yabaki sa oti. Era na sasagataka na kena dau ke rawa ni sivia, e leqa ni vakaiyalayala na veika e qaravi taumada kina (Bromberg & Bertness 2005). Ena gadrevi me vakasaqarai tale eso nai tukutuku ka volai taumada ni se bera ni yaco mai nai walewale ni vakasokumuni tukutuku me vaka na veitaba eso mai na maliwa lala.

Na veimape kei nai yaloyalo ni veigauna sa oti yani ena veivuke sara vakalevu ka ni tauri ena gauna se bera mai kina nai walewale vou ni vakasokumuni tukutuku me vaka nai taba mai na maliwa lala. Nai walewale oqo ena okati talega kina na veiririko eso e sotavi ena veicakau, veimatasawa kei na veidogo. Nai walewale makawa eso ni vakasokumuni tukutuku kei na kena vakaivolataki e vakaraitaka na veiveisau ni veicakau ena yatu *Florida Keys* ena 240 na yabaki sa oti (McClenachan et al. 2017), veikau mai *British Columbia* me dua na senitiuri (Costa et al. 2020), erau vakatauvatani na veimape oqo kei nai taba ka tauri mai na maliwa lala me veidutaitaki keina veisau eso e sotavi ena *California oak savannahs* ni sivia e dua na senitiuri (Whipple et al. 2011). Na veimape eso ni Pasivika era vakarautaka taumada na vavalagi e vakatara na kena vakadikevi na veiveisau eso era lako curuma nai yaubula duidui eso, ka sega ni okati kina na veitukutuku eso esa vakaivolataki oti tu ka ra veisemati vata. O keda nai taukei eda veisemati vakavoleka kei ira nai yaubula eda bula veitikivi, ka okati kina na veicakau kei na veidogo talega eso.

***Na kena mapetaki na loma ni vanua kei na wasawasa***

O Viti edua na yanuyanu ena wasa Pasivika. O ira na kenai taukei era bula tu kina me rauta e 3500 na yabaki, era veisemati vakavoleka sara kei waitui ka vaka talega kina na vanua mamaca. O Viti e tiko ena seda, ena vuku ni rawaka vakailavo, veivakatorocaketaki vei ira na dauni bisinisi lelevu kei na veitosoyaki ena vuqa na gauna sa oti yani (Cochrane 2017). E dina ni rau a raica taumada o *Tasman* (in 1643) kei *Cook* (in 1784) na yanuyanu o Viti, ia e qai toqa na kena mapetaki na yanuyanu o Viti na turaga ni vavalagi o William Bligh, ni oti na nodra veiravuravu ena H.M.S. Bounty ka takosovi Viti ena nonai golegole i Indonesia (Bligh 1792). E kuria na kena vakaivolataki na *United States Exploring Expedition* ena 1840 (Wilkes 1845, Fig. 1) e tokona talega na cakacaka oqo na *U.S. colonial interests* (Smith 2013). Ena gauna e qai tauri kina na vakaukauwa na qele vakaitaukei na 1874, na *British Admiralty* e sa tabaka oti tu na kena mapetaki na veicakau me vukea na nodra veitosoyaki vakamatau na waqa lelevu. Nai curucuru ni waqa e Suva e vakaivolataki ena 1882 (Seemann 1862). Na kena torocake na bula ni politiki kei rawaka vakailavo ena vanua o Suva, e vakavuna na nodra toso mai vakalewe levu na lewenivanua ena koro turaga, ka tubu cake talega kina nai wiliwili enai ka 20 ni senitiuri (Ward 1959).

Ena yabaki 1900s e kunei na kena totoka na kedrai tuvaki na veimataqali cakau kei nai veidogo (Sparling 1923). Davis (1918) ena vanua o Suva ka valamacalataki me vaka oqo : “*Among other associates of the lagoon corals are sea urchins with long black spines like knitting needles, large dark blue starfish, slowly crawling reddish -brown sea - cucumbers, and many kinds of fish of varied hues … which swim about at ease among the coral branches. … The most remarkable are the gigantic clams, one or two feet in length, which live embedded in the reef flat, hinge-line down…”* Ni oti tale e 20 na yabaki e vakamacalataka o Barrett (1935) ka vaka, *“The crystal-clear waters of the lagoons of Fiji Harbour contain many bright-hued fish and coral formations of a fairy-like beauty.”*

Nikua e laurai ni volekata e dua nai katolu ni lewenivanua era vakaitikotiko volekati Suva, ka vakavuna na levu ni revurevu era sotava nai yaubula ena vanua oqo (Dacks et al. 2020). Ni mai cava nai ka 20 ni senitiuri e laurai ni vakasavasavataki na boto ni wasaliwa (Penn 1983), na vei volau ni ta waqa era vakacacana nai yaubula ena noda wasawasa (Davis et al. 1999) ka okati talega kina na nodra vakarusai na veicakau dromu eso ena vuku ni wai ca mai vanua (Lal et al. 2018).

E duatani na mataqali revurevu e sotava na vanua o Savusavu mai Vanua Levu, ni vakatauvatani kei na levu ni revurevu e sotavi tiko i Suva. E tauyavutaki na tauni o Savusavu ena 1969, na veivure ni waikatakata lalai eso e kauta mai na kena tauyavutaki eso na veitikotiko lalai volekata (Dana 1849). James Dwight Dana (1849) ka dui tu na kenai vakamacala me vaka, *“A plain rising with a gentle slope from the water extends back from the shore about three-fourths of a mile, and then passes into the steep declivities of a high broken ridge, running nearly parallel with the coast. Patches of grass, and much low, dense shrubbery, cover the plain near the springs; and some distance to the right and left, large coconut groves throw some little beauty into a scene otherwise unattractive.”*

E kilai o Savusavu ena waikatakata vure kei na niu, ka okati talega kina nai curucuru ni waqa e titobu, ka rawa talega ni vakaruru kina na *U.S. Pacific fleet* ena gauna ni karua ni valu levu (Inskip 1937). E dina sara ni kilai o Savusavu ena niu (copra), e na vuku ni veivakatorocaketaki eso, io e laurai ni saravanua e gaunisala ni rawaka tiko vakailavo (Graci & Vliet 2020). Na veivesau qo e okati talega kina na kena vakayagataki na veidogo eso me tara kina na bure ni vulagi (J. Drew pers. obsv.) kei na vuqa tale na veivakatorocaketaki ena vanua oqo (Prasad & Raturi 2019). Ni vakatauvatani kei Suva, na vakatoroicaketaki ni kena veimatasawa e sega ni vu mai na levu ni waqa se tubu ni wiliwili ni lewenivanua. Oqo e dua na vuna ka bibi me dau kilai taumada na veitukutuku makawa me vakataki Savusavu, ka ni rawa ni vakatakila na vegaunisala eso erawa ni da dau vakavuna na tamata ena vuku ni revurevu eso era sotavi me baleta nai tuvaki ni veiyaubula.

Matai ni wasewase

Keitou na vakayagataka e so na veitukutuku kei na mape ka railesuva nai tuvaki ni veicakau kei na kena yaubula e Suva kei Savusavu me laurai na veiveisau eso era lako curuma. Nai iTaukei era vakararavi ena veidogo kei na veicakau ena vuku ni kana ena veisiga, rawaka vakailavo kei na veika bulabula eso e solia na veiyaubula oqo (O’Garra 2009; Sangha et al. 2019). Na kena vakatakilai na veiririko eso e vakavuna na nodra vakacacani na veiyaubula, ena vukea na kena tarai cake nai walewale e matau ni nodra taqomaki nai yaubula oqo. Ena vukea talega na neitou kila nai tuvaki ni veiyaubula oqo ena gauna era tuvaki vinaka kina. E vakayagataki nai walewale qo me veidutaitaka na veivakatakilakila eso me vaka na veilase eso, tuvaki ni lase kei na veidogo kei na veimatasawa talega. Na veivakatakilakila kece oqo e vukea na neitou kila nai tuvaki ni rua na tauni oqo kei na veiveisau eso e rau lako curuma ena loma ni 160 na yabaki sa oti. E laurai na levu ni veisau ena loma ni 160 a yabaki, o Suva e laurai kina na revurevu ni veivakatorocaketaki ena vuku ni kena vakacacani na veicakau (e.g., Lal et al. 2018), ka namaki sara tiko vakalevu na veika qo ena vo ni gauna ena vakadikevi kina. E lailai na revurevu mai Savusavu, ni sega ni levu nai wiliwili ni tamata. Ia ena vakilai na lailai ni veidogo (Lal 1990). Na I tukutuku dau vakasokumuni taumada ka vakaivolataki vinaka ena vukea na veidutaitaki ni veicakacaka ni mamaroroi se tataqomaki e sa yaco tiko mai kei na veituvatuva matau me na salavata. Ena vukea talega na nodra cakacaka na kena dau ena ena kena vakavinakataki ka vakarabailevutaki na veitukutuku eso sa vakasokumuni taumada (Bao & Drew 2017), na veitukutuku oqo ena vukei ira nai lewenivanua mera nanuma na veika eso e vutuniyau kina na veicakau ena noda vanua.

**Kena walewale**

E railesuvi nai tukutuku mai na 150 na yabaki sa oti ena vuku ni veiveisau ni veicakau kei na veidogo mai na toba o Suva kei Savusavu *(Fig. 2).* E vakatauvatani na veitukutuku vakasokumuni eso kei na kena mai na *British Admiralty, U.S. Exploring Expedition, U.S. Army* kei na *Fijian Lands kei na Survey Department*, e okati talega kina na veimape eso mai na *U.K. Hydrographic Office, National Library of Australia, State Library of New South Wales,* kei na *Bodleian Library* mai na *Oxford University*. Na kena vakasaqarai, railesuvi ka biu vata na veitukutuku oqo e kumuni mai na sivia e 50 na kena veimataqali lavelave eso, me vakayagataki ena porokaramu oqo. Na veivolatukutuku e kumuni ka vakayagataki me veidutaitaka nai tukutuku, e sega ni kau mai na dua walega na vanua, ia e vakatakila e dua na vanua rabailevu ka ra bula vinaka na kena veicakau kei na veidogo. Na kena tabaki tale e muri e vakatakila vakamatailalai na veicakau kei na lase eso kei na veisau era lako curuma.

Karua ni wasease

Ena neitou kila, na veilavelave e vakayagataki e vakatakila nai walewale duidui e vakayagataki (*sampling efforts)* se vakatakila na veiveisau lelevu e yaco ka yavutaki mai na macala ni vakadidike. Nai lavelave ka yavutaki mai nai walewale e tautauvata na kena vakasokumuni mai, e sega ni qai vakayagataki me kakua ni veivakacalai. Na vakatulewa qo e yavutaki mai na macala ni vakadidike ka toqai tu enai lavelave. Na kena toqai na tiki ni siga e tabaki kina na veitukutuku se lavelave oqo, e vakatakila na gauna e railesuvi kina na volaitukutuku oqo.

Table 1: Historical charts of Suva and Savusavu Harbor used in this manuscript, including citation information and analyses included. Note the scale for 1840 Suva is an approximation as true scale is not provided on the chart.

| Suva Harbor | | | | | | |
| --- | --- | --- | --- | --- | --- | --- |
| Title and Author | Date | Scale | Publisher / Creator | Library, Archive, Source of Reproduction | Coral Area Analysis | Mangrove Length Analysis |
| Suva Harbour south side of Viti Levu, 1840, C. Wilkes | 1840 | Approximately 1:50,000 | U.S. Exploring Expedition | State Library New South Wales | Graphed, not Analyzed | N/A |
| Fiji Islands: Viti Levu: Nukulau Island to Namuka Island Including Lauthala, Suva and Namuka Bays, L.S. Dawson | 1875 | 1:24,370 | UK Hydrographic Office | Bodleian Library at Oxford | Included in Analysis | N/A |
| Fiji Islands: Viti Levu: Suva Harbor (Tomba Ko Suva), J.W. Combe | 1898 | 1:12,150 | UK Hydrographic Office | UK Hydrographic Office | Included in Analysis | Included in Analysis |
| Fiji Islands: Viti Levu: Nukulau Island to Namuka Island Including Lauthala, Suva and Namuka Harbs. New Edition Dec. 1914.  L.S. Dawson | 1914 | 1:24,370 | UK Hydrographic Office | Bodleian Library at Oxford | Not Analyzed | Included in Analysis |
| Fiji, Viti Levu, Suva & Lower Rewa, Tourism, 1934, 1:63 360 | 1934 | 1:63,360 | Fiji Lands and Survey Department | Australian National Library | Included in Analysis | Included in Analysis |
| Fiji, Viti Levu, Suva, Series: X751, Sheet 67, 1943, 1:62 500 | 1943 | 1:62,500 | U.S. Army Map Service | Australian National Library | N/A | Included in Analysis |
| Fiji - Suva [cartographic material] / published (with the help of various New Zealand agencies) by the New Zealand Department of Lands and Surveys. | 1945 | 1:12,672 | Fiji Lands and Survey Department, New Zealand Department of Lands and Survey | State Library New South Wales | Included in Analysis | Included in Analysis |
| Fiji Islands: Viti Levu: Suva Harbor | 2019 | 1:12,000 | UK Hydrographic Office | UK Hydrographic Office | Included in Analysis | Included in Analysis |

| Savusavu Bay | | | | | | |
| --- | --- | --- | --- | --- | --- | --- |
| Title and Author | Date | Scale | Publisher / Creator | Library, Archive, Source of Reproduction | Coral Area Analysis | Mangrove Length Analysis |
| Fiji Islands: Vanua Levu: Savu Savu Bay, H. Barrack | 1876 | 1:177,100 | UK Hydrographic Office | Bodleian Library at Oxford | Graphed, not Analyzed | N/A |
| Fiji Islands: Vanua Levu: Savusavu Bay, United States Hydrographic Office, 3rd edition, No. 2853, 1921 | 1880 | 1:100,920 | UK Hydrographic Office | U.C. San Diego Library | Included in Analysis | Included in Analysis |
| Fiji, Vanua Levu, Savusavu Bay West, Series: X754, Sheet 11, 1962, 1:50 000, & Fiji, Vanua Levu, Savusavu Bay East, Series: X754, Sheet 12, 1966, 1:50 000 | 1966* | 1:50,000 (over 2 charts) | Directorate of Overseas Surveys | Australian National Library | Included in Analysis | Included in Analysis |
| Fiji Islands: Vanua Levu: Savusavu Bay | 2018 | 1:50,000 | UK Hydrographic Office | UK Hydrographic Office | Included in Analysis | Included in Analysis |

E vakayagataki na *ArcGIS Desktop 10.8.0 keina R Studio Version 1.3.1073* me veidutaitaka na duidui ni veitukutuku e vakasokumuni mai, e okati talega na kena veidutaitaki kei na veitukutuku eso mai na *WGS 1984* ka salavata kei nai walewale eso ka vakamacalataki tu ena *McClenachan et al*. (2017) and *Costa et al.* (2020). Oqo na va na veimataqali walewale e vakayagataki na *Projective and First-Order Polynomial Transformation* e vakatauvatana na veika me veiraurau ka yavutaki ena *RMS error (each map < 0.001 RMS error).* Na veitukutuku eso e vakatauvatani ena vakasokumuni tukutuku oqo e kovuta e dua na vanua lailai ga, ka veidutaitaki ena *RMS error*, me rawa ni vakalailaitaka na kena sega ni veidonui eso nai tukutuku (Jenny and Hurni 2011).

E vakayagataki na veivakatakilakila e so me vakatakila na kena vakarautaki na veiveisau eso ena veicakau, veidogo kei na veimatasawa eso. E vakayagataki na veiwalewale eso mai na kena e lavetaki taumada mai liu, kei na kena vakayagataki nai walewale vou, ka sa qaravi tu ena gauna oqo. Na veilavelave se tukutuku eso e toqai ni se bera na 2018, e okati talega kina na kena droinitaki na veicakau kei na veidogo. Era vakatakilai na veicakau ena *(Fig. 3)* e vakaraitaka na kena vakatakilakilataki na veicakau. E dina ni duidui na veika eso e ra cauraka mai na veimataqali mape, ia e vakadeitaki na kena tabaki me rawa ni ra vakatauvatani. Na veivakatakilakila eso e vakayagataki kina nai walewale oqo ni *ArcMap Conversion*. E vakayagataki eso na matanifika (17.32 m) me veidutaitaki na veiduidui vakafika eso ena veimape kei na kedra dui vakarau me vakatauvatani kei na veicakau lalai (300 m^2^). E dua nai walewale ni veiqaravi ka vakatokai me *Spatial Analyst* e vakayagataki kei na kena veivakatakilakila eso me rawa ni wasea na veivanua eso era bula kina na veilase eso kei na veivanua e sega kina na lase. Na veiyabaki era dau veitudaitaka na kena dau veiveisau era lako curuma na veicakau, ena vuku ni kena levu kei na vanua eso e kovuta. E vakayagataki nai walewale se yaragi ni vakadidike ka vakatokai na *CA (patch area), CORE (core area, or the area of each patch which is not edge), NP (number of patches), PD (patch density, or the number of patches per the total area, standardized for comparison), PERIM (length of edge), and ED (edge density, or the total edge length per the total area, standardized for comparison) from R package landscapemetrics (Hesselbarth et al. 2019) to compare raster metrics.*

**Katolu ni wasewase**

E vakatakilai talega na veidogo mai na veivakatakilakila eso kei na matasawa e ra volekata. Na balavu ni matasawa e na vakatakila na veiveisau ni veidogodogo. E vakaisosomitaki na veimatasawa kei na nuku ena veivakatorocaketaki lelevu era sa yaco mai, ni ra veidutaitaki na veivakatorocaketaki oqo kei na veimatasawa, ka laurai ni sega sara na veidogo e bula rawa kina. E vakatakilai na veivakaraitaki qo enai lavelave vakarautaki, ka sega ni vakatakilai kina na veigusuniwai. Na veivanua eso ka sega ni veiwekani kei na veidogo se veimatasawa, se veivanua eso era sotava veiveisau eso ena vuku ni revurevu eda vakayacora na tamata e vakatakilai tani mai nai lavelave vakarautaki ka okati talega kina na vanua veicoco, kei na vanua veivatuvatu. E ra vakarautaki na duidui mai na veivanua duidui qo me vakatakilai enai lavelave vakarautaki.

Na kena vakatauvatani nai tukutuku matailalai ni veilase kei na veidogo ena veilavelave duidui eso e vakarautaki e sega ni vakatakilai ena *(Table 1)* mai cake. E vakatauvatani talega na veivanua lase kei na veidogo eso kei na veivanua tale eso me veidutaitaki na veika e so e vakayali se vakalesui mai kina. Nai lavelave vakarautaki me baleti Suva e laurai ni tautauvata tu ga ena veigauna, ka duatani sara nai tukutuku e vakaraitaki mai Savusavu ni gauna e qaravi kina na veidutaitaki ni tukutuku mai na vanua e rua oqo. Nai matai ni vakadidike e vakatabakidua ga ena tauni o Savusavu kei na kena veicakau e volekata ka rauta ni *(~3625 hectares)* na kena levu ni vakatauvatani kei Suva. Na kenai karua e rauta ni 112,000 na eketea. E dina ni levu na duidui e kune ena mape me baleti Suva kei Savusavu, ia e tautauvata nai vakatagedegede ni veika se veitukutuku e cauraki me baleta na vanua e rua oqo. E na yabaki 1996 mai Savusavu e rua na mape tautauvata e cokoti vata me dua ga ena vuku ni veika e cauraki tiko ena loma ni mape e rua oqo. Ka tabaki ena 1962 mai na ra ka tabaki ena 1966 ena tokalau.

E dua nai walewale ni veiqaravi ka vakatokai *chi-square tests* e vakayagataki me vakatauvatana na veicakau ena vanua ruarua. E vakayagataki na *linear model*, me vakamacalataka na veiwekani ni veidogo, veimatasawa kei na yabaki. Ni se bera ni vakayacori nai walewale oqo e vakadeitaka na *Shapiro-Wilk Test prior (‘stats’ R Core Team 2020)* na veitukutuku oqo. E vakatauvatani nai tukutuku vakasokumuni ni veidogo ni tolu na yabaki mai Savusavu kei na walewale na chi-square, ena vuku ni lailai ni vanua e sabolotaki.

**Na macala ni vakadidike**

Nai tukutuku e vakaraitaki ena *Table 1,* e kau mai na walu nai tukutuku vakarautaki kei na mape me baleti Suva kei na va nai tukutuku vakarautaki kei na mape me baleti Savusavu ka kovuta na loma ni 180 kei na 150 na yabaki sa oti. E vitu nai lavelave e vakamatatataka na veitukutuku matailalai me baleta na veilase, ia na mape ni 1914 me baleti Suva e droinitaki tu kina na lase mai na walewale ni sabolo ka vakayacori ena 1898, ka vuna na kena sega ni vakayagataki kina na mape oqo. Na veitukutuku lavelave makawa me baleta na vanua e rua oqo, e sega ni vakamatatataka mai nai tukutuku eso ni lase ena gauna koya (see Lukas 2014), ka sega ni matata vinaka na veiduidui eso ena vuku ni kila vakadroini ena gauna koya, ka okati talega kina na mape vakarautaki me baleti Suva (see Lukas 2014). Na kena vakamatatataki mai nai tukutuku matailalai ni veiveisau eso e sotavi ena vuku ni lase kei na veicakau e tekivu mai na 1875 ki na 2019 I Suva (n = 5), kei na 1880 ki na 2018 (n = 3) i Savusavu.

E dina ni sega ni veisau sara vakalevu nai tuvaki ni lase i Suva ena gauna e vakayacori kina na vakadidike oqo, ia e toso ga vakalailai na batini cakau ena maliwa ni yabaki 1875 ki na *2019 (χ^2^, p < 0.001)*, ia e duidui na kena bibi ena gauna vata ya, ka veisau talega na kenai tuvaki *(χ^2^, p = 0.88)*. E tubu cake na vanua e kovuta na lase ena 8% *(+40 hectares, χ^2^, p < 0.001)* ka tubu cake talega na kena veivanua eso me *(8%, +34.7 hectares, χ^2^, p < 0.001).* Na kena levu taucoko ni vanua duidui eso ka vakasokumuni mai na kedrai tukutuku mai nai lavelave ni 1875 ki na 2019, ka sega ni laurai kina vakalevu na duidui eso e *(χ^2^, p = 0.72, p = 0.99 respectively).*

**Ka va ni wasewase**

Na veicakau e tiko ravita na tauni mai Savusau e sotava vakalevu revurevu ni vakacaca *(Fig. 4; χ^2^, p < 0.001)* ka okati talega kina na toba e Savusavu, e yali talega kina e rauta ni 2933 eketea ni veicakau mai na yabaki 1880 ki na 2018. E laurai talega ena *(Fig. 4)* na duidui ni bati ni veicakau ena gauna yabaki vata oqo, ka vu mai oqo ena lailai ni vanua era bula kina na veilase kei na kena tubucake na 90% na veivanaua tale eso *(Fig. 5; χ^2^, p < 0.001).*

**Ka lima ni wasewase**.

E okati talega na kena vakadidike oqo, me baleta na veicakau, na kena vakadikevi na veidogo kei na veimatasawa i Suva kei Savusavu. E vakayagataki e vitu nai lavelave me baleti Suva me vakatekivu mai na yabaki 1898, kei na tolu nai lavelave mai Savusavu me vakatekivutaki mai na yabaki 1880, se vakatakilai nai tukutuku oqo ena (Table 1).

Ena yabaki 1898 e kunei ni rauta e 79% na veidogo ena veimatasawa i Suva, e mai veisau ena yabaki 2019 ni sa laurai ga e 35% *(Fig. 6).* Ni vakatauvatani kei na veimatasawa ka tiko volekata na veitauni e tubucake mai na 9% ki na 58%. Na tubucake ni wiliwili oqo e kunei ena vanua e tara kina na ba ni ua Ena yabaki 1943, na veimatasawa tale eso e tautauvata tu ga me 17% na kena lailai na veidogo e sega ni veiganiti kei na yabaki *(lm, -0.299, p = 0.13, adjusted r^2^ = 0.48),* ni vakatauvatani kei na tubucake ni wiliwili ena tauni e veiganiti vinaka *(lm, 0.003, p = 0.056, adjusted r^2^ = 0.64).*

Ni veidutaitaki kei na tauni mai Savusavu, na veidogo e dua tu ga ena *17% (3.7 % SE, χ^2^, p = 0.09)* oqo ena matasawa ka voleka mai tauni. Na tara ni ba ni ua kei na veisala e so e vakayagataki me tarova na sisi ni veimatasawa e toso cake ena yabaki 1966 mai na 7% ki na 51%, ni vakatauvatani kei na veimatasawa tale eso *72% ki na 39% (χ^2^, p < 0.001; Fig. 6).* E vakilai talega nai veisau oqo ena toba mai Savusavu ena kena tubu cake na veivakatorocaketaki ni tara ba ni ua mai na 3% ki na 40% ena yabaki 1960. Ni da toso mai vakayawa mai tauni e kunei vakalevu sara na veidogo ena veimatasawa (rauta ni 40%, 2.5% SE), ka sega sara ni veisau vakalevu mai na yabaki 1880 kei na 2018.

Ka ono ni wasewase

**Veitalanoa/Veivosaki**

Na cakacaka keitou qarava e vakaraitaka na veiveisau eso e sotavi, ena vuku ni yaubula kei na revurevu ni veivakacaca eso ni veivakatorocaketaki lelevu se lalai eda vakayacora na tamata e na veimatasawa i Suva kei Savusavu. E dina ni daumaka na bula ni lase i Suva, ia ni gauna e vakacacani kina na veidogo, e lutu sobu nai vakarau ni yaubula era bula era bula veitikivi ena veimatasawa me rauta e 50 na pasede. E levu sara na kena vakacacani na veicakau kei na lase mai Savusavu. E dina ni ra yavalati na veidogo ena vuku ni veivakatorocaketaki ena veimatasawa mai Savusavu, e kunei votu na tubu cake ni revurevu ni vakacaca ena vanua e rau veisemati kina na vanua mamaca kei na waitui. Ni vakasomukuni vata na veitukutuku kei na macala ni vakadidike eso e qaravi e Suva kei Savusavu e laurai ni na kena veimatasawa e se vakacacani sara ena dua nai taba gauna ena vuku ni veivakatorocaketaki ni vakaqarai lavo. Na macala ni vakadidike e mai qaravi, e vakatakila na rabailevu ni nodra bula vakaveiwekani ka veisemati nai yaubula duidui eso, kei na kena gadrevi na kena veidutaitaki nai tukutuku me vakaqaqacotaka na veivakanananu kei na veitukutuku eso mai na veigauna sa oti yani.

Na levu ni tukutuku me baleta na kena vakacacani na veicakau i Suva (Naidu and Morrison 1994), e vakadeitaka na veitukutuku kei na lavelave makawa eso ni curucuru ni waqa i Suva e vakatakila eso na veicakau vinaka, ka vakaraitaka talega, na kena tubucake na nodra veitosoyaki ni lewenivanua mai na koro vakaviti kina koro vakavalagi e sega ni vakilai vakalevu na kena revurevu ena veivanua oqo, sega ni okati kina nai tuvaki ni lase, na de ni qele kei na kena vakacacani na lase ena vuku ni sivia ni katakata ni waitui. Nai walewale se yaragi ni veiqaravi e vakayagataki ena vakadidike oqo, e sega ni rawa ni vakatakila mai nai tuvaki ni lase ena dua na gauna, ena sega talega ni vakadeitaka na kenai tuvaki kevaka e se rawa tikoga ni taqomaka se maroroya na veimatasawa. Na vakdeitaki ni tuvaki ni veivanua e bula kina na lase i Suva ena vakatakilai mai ena veimataqali lase duidui era bula kina me vaka na vulavula ni lase na *Porites spp.* (Goberdhan & Kininmonth 2021), edua na mataqali lase erawa ni bula donumaka na veivakacaca eso ni vakatauvatani kei na kena mataqali era bula mai Savusavu. E vakadinadinataki ena vakadidike ni ravuta talega vakalalai na nodra bula mai na veilase vovou na de ni qele kei na vakabenubenu eso (Lal et al. 2018), ka vakaraitaka ni tiko eso na veicakau e rawa ni ra bula donumaka eso na veivakacaca eso. Ni vakatauvatani kei Savusavu, ena vuku ni revurevu e tarai ira na lase kei na veicakau, e laurai ni yaco na veika oqo vei ira na cakau era tiko veitikivi mai na yasani tauni, ka dau nanumi ni dau tuvaki vinaka na veicakau era tu kina (see Goberdhan & Kininmonth 2021). E vakilai na tubucake ni katakata ni wai mai Savusavu ena 2000, ni rauta ni 40% na mataqali lase ka vakatokai me *scleractinian corals* era mate kina (Cumming et al. 2006). Okati talega kina nai vakadinadina ni raramusumusu ni lase ka vakatakila ni veiveisau na nodra bula nai yaubula kei na vanua era bula kina mai Savusavu ena loma ni 50 – 100 na yabaki.

Na revurevu ni veivakacaca e laurai i Savusavu e vakaraitaka na kena sega ni qarauni se vakayagataki vakamatau nai yaubula ena loma ni vanua. Na veivakadidike e qaravi taumada e vakatakila na maucokona ni veiwekani ni yaubula kei na vanua era dui bula kina (Bellwood & Hughes 2001; Knowlton 2001). Na kena vakacacani se vakayali na veivanua eso era bula kina na yaubula e vakavuna na kena lalai nai vakarau ni ika kei na kedrai wiliwili, okati talega kina na kena vakacacani na veilase eso kei ira na yaubula duidui era bula veimaliwai vata (Caley et al. 2001; Bonin et al. 2011). Na kena tubucake na vakacacani ni veivanua era bula kina na lase e laurai ni ra tubu talega mai eso na lase vovou (Bonin et al. 2011). Na batilili era bula talega kina na ika era bula ena veivanua e nuku, ka rawa ni duidui mai na vo tale ni veivanua eso (Ault and Johnson 1998; Friedlander and Parrish 1998) e vakadinadinataki ni na veibatilili e sega ni levu na lase era bula kina (Sambrook et al. 2016). Na kena levu kei na duidui ni veimataqali vanua eso era bula kina na lase ena vukea talega na kena tubucake na veimataqali veiyaubula duidui eso era na bula mai kina, e dina ni se bera ni vakadinadinataki sara na tikina oqo, ia na kena tubucake ka sautu na veiyaubula oqo erawa ni ra vakavuna na vei ika eso (Sambrook et al. 2016). Na kena volaitukutukutaki na yali se mate ni ena dua na gauna ena rawa ni vukea na kena vakadikevi lesu nai tuvaki ni vanua ena gauna qo me vakatakila mai na vuna ka tauyavutaki edua nai walewale matau me rawa ni vakacokotaki kina se vukea na kena maroroi ka taqomaki talega (e.g., Bromberg & Bertness 2005). Na kena tauyavutaki edua nai walewale matau ni nodra maroroi se taqomaki na veivanua duidui eso, ena vukea na kena vakalailaitaki na vakacacani se vakayali na nodrai tikotiko nai yaubula duidui oqo.

E laurai na levu ni kena vakacacani ka yali na veidogo e Suva, ka vakadeitaka nai tukutuku ni kena vakacacani na veidogodogo ena Pasifika ka vakabibi e Viti (Lal 1990). E laurai ni veicalati nai tukutuku ni kena vakacacani na veidogo kei na yabaki ni rau veidutaitaki, sega ni veivakurabuitaki nai tukutuku oqo, ni veidutaitaki kei na kena vakacacani talega na veikau eso. Na kena vakaveiwekanitaki na yali ni veidogo kei na yabaki e vakadeitaki mai na yabaki 1891 me yacova mai nikua. Na duidui ni kena veiveisau na veimatasawa e Suva kei Savusavu e vakaraitaka na duidui ni tuvaki ni vanua. Ena vuku ni tubucake ni veivakatorocaketaki kei na kena vakarabailevutaki na bula i Suva, e vakavuna na kena sa yali yani na veimatasawa kei na veidogo vinaka, ni vakatauvatani kei Savusavu, e sega soti ni vakacacani sara vakalevu na veidogo kei na veimatasawa ena vuku ni levu ni qele eso era sega tu ni vakayagataki. Ena vuku ni kena lade na gauna e vakasokumuni kina nai tukutuku nai na yabaki 1880 kei na 1962, e sega ni vakaraitaka se rawa ni vakadinadinataka kevaka era vakacacani na veidogodogo ena vuku ni teitei ena 1896 (Lal 1990). Ia na macala ni vakasokumuni tukutuku e e vakadeitaka na kena lailai na veidogo ena yabaki qo ka rauta me (43% ki na 35%) ka gadrevi na kena vakacokotaki lesu na veidogo oqo. E dina ni rawa ni vukea na vakadidike sa mai qaravi nai tuvaki ni matasawa ena dua na gauna sa oti yani, ia ena sega ga ni vakadeitaka na levu ni veidogodogo e vakacacani ena dua na gauna. Me na tomani na kena vakasaqarai nai tukutuku me baleta na kena vakacacani na veidogodogo ena dua na gauna makawa, ena vakayagataki nai taba mai na maliwa lala me dikeva na veivanua oqo ka tokona talega na vakadidike ka vakayagataki kina nai taba eso me vakadinadinataka na veiveisau eso ena veidogo mai na yabaki 2000 (Cameron et al. 2021).

Na kena yaco mai na veivakatorocaketaki lelevu eso ena veimatasawa kei na veidogo, e vakatakila na noda kuitaki ena veivakananau eso e kauta mai na gauna vakakoloni. Nai vakadinadina e laurai nai *taba (Fig. 1, Supporting Information)* na kena veisau mai na veimatasawa kei na veidogo vinaka, ki na ba ni ua, kei na gaunisala ka tekivutaki na veivakatorocaketaki ena yabaki 1914 i Suva, enai ka 20 na senitiuri, ka dua na revurevu ni veivakatorocaketaki ena koro vakacakacaka, ka vakadredretaka na kena tarovi na sisi ni veimatasawa (e.g., Thampanya et al. 2006), ka okati talega kina na kena yali na veiyaubula eso era bula ena vanua oqo. Na veidogo e levu sara na kena yaga, e tarova se vakamalumutaka na ua gunu kei na ua loka (Granek and Ruttenberg 2007), e nodra sususu na veimataqali sasalu ka vurevure ni rawaka vakailavo me vaka na qari kei n avuqa tale (Carney 2017), vukea na kena vakabulabulataki na veiyaubula eso era bula tiko kina ena vuku ni veika vivinaka e vakarautaka (Feller et al. 1999), e tavulona na cagi (Alongi 2014) ka vanua era susu ka bula talega mai kina na vei ika duidui eso (Mumby 2006). E salamuria na vakacacani ni veidogo na lailai ni rawaka vakailavo ena vuku ni veisasalu eso era bula, na kena vakayagataki eso nai walewale e saulevu cake me tarova na ua gunu kei na ua loka, na kena vakayagataki me tara kina na vale dina ga ni veisosomitaki na kena musu na veidogo me vakayacori kina na teitei se susu ura (see Lal 1990). Me kuria nai tukutuku sa toqai toka mai cake, na veidogo e sega soti ni vukea na kena nodra vakawa na ika lelevu ni veicakau (Brooker et al. 2020). E sa na yaga me dau veitalanoataki vakavinaka kei ira na lewenivanua na veivakatorocaketaki eso ena vakayacori ena veidogo kei na veimatasawa. Me na dau vakayacori vakamatau me rawa ni sotava na revurevu ni tubucake ni yalayala ni wai era sa sotava tiko na lewenivanua ena gauna qo ena Pasifika.

Na kena vakatoroicaketaki na veimatasawa kei na veidogo e rawa ni vakavuna na kena bula mai na veiyaubula eso era vulagi ena veivanua oqo, ka na vakavuna na ririko ena veilase kei na yaubula tale eso era bula veitikivi (Goldberg & Wilkinson 2004). Nai kelekele ni waqa e Suva e ririkotaki sara vakalevu ni vakatauvatani kei Savusavu ena vuku ni levu ni waqa era gole tiko mai kina, okati talega kina na kena dauvakasavasavataki na boto ni waqa lelevu eso, ia na revurevu ni kena vakacacani na veimatasawa e laurai ena vanua ruarua e qaravi kina na vakadidike. I Viti, e vica walega na sasalu vulagi e sa volai ka kilai rawa me vaka na *Mytilopsis sallei* (Bax et al. 2002), *Ostrea edulis* (Bromley et al. 2016), *Gracilaria edulis,* *Sargassum polycystum* (Charan et al. 2017), and *Kappaphycus* spp. (Sulu et al. 2003) e rawa ni vu mai qo ena lailai ni kila vakavuku ka okati talega kina na kena lailai se bera ni vakayacori e dua na kena vakadidike. E sega sara ni matata vinaka nai tuvaki ni veiyaubula vulagi eso ena veikelekele ni waqa, ka dravudravua sara vakalevu na kena kilai na veitukutuku oqo (Hutchings et al. 2002), oati talega kina na Pasifika, me tomani na kena vakadikevi me rawa ni vukea na kena kilai na gaunisala eso e curuma tiko mai kina na noda veidogo kei na veimatasawa.

E levu sara na bolebole e sotavi ena vuku ni kena biu vata na macala ni vakadidike eso, ka ni dua nai wase levu ni cakacaka ena vakatautaki tiko vei ira era vakasokumuna taumada nai tukutuku mai liu, ka dua talega nai walewale vinaka ka rawa ni vukea na kena maroroi ka taqomaki na wasawasa (McClenachan et al. 2015; Thurston et al. 2015). Na veilavelave makawa eso e vakatakila e so nai tukutuku makawa, ia ena vuku ni kena sega ni matata sa ra vakavinaka na kena vaivolataki, e vuna na kena sega ni rawa ni vakayagataki eso nai tukutuku e salavata tiko (Lukas 2014). E levu nai walewale kei na kena vakaivolataki na mape kei na lavelave eso, ni vakatauvatani kei nai lavelave e vakarautaki me baleti ira na mataivalu kei na veitosoyaki eso e sega sara na kena duidui (Vellend et al. 2013). Dina ni lavetaki enai naki e tautauvata ga, ia na gauna e droinitaki kina na veilavelave se volatukutuku oqo e vakatakila ga na veika eso e gadreva o koya e vakarautaka mai, ka sega ni veiraurau kina eso nai tukutuku e talaucaki kina. Na kena vakatulewataki se veisautaki eso nai tukutuku ena gauna e railesuvi kina e vakavuna na kena vakacalati eso nai tukutuku. Nai lavelave ka vakatokai na *nautical charts* e laurai ka veidutaitaki vakavinaka ni sega ni rawa ni vakatakila vinaka na duidui ni lase bula kei na lase mate ni laurai mai na dela ni wai. Ena kena gadrevi me biu vata nai lavelave duidui eso enai walewale ni cakacaka ena gauna qo ka vakatokai na *GIS layers*, e laurai na veisau ena veilavelave oqo. Na levu ni veimataqali veisau e laurai ena nodra bula na lase, veidogo kei na veivakatorocaketaki era sotava ena yaga me dua tale na kena vakadikevi ka laurai vakavinaka na veika eso era lako curuma tiko. E sa namaki tiko na veisivi ni veitukutuku eso ena vuku ni lailai ni tukutuku vakarautaki ena veimape (Lukas 2014).E dodonu me dau veitudaitaki ka railesuvi na macala ni vakadidike oqo ena vanua e vakayacori taumada kina. Na macala ni vakadidike me baleta na vanua era bula kina na lase kei na veiveisau era lako curuma erawa ni vakadeitaki mai na levu ni de ni qele ka kumuni mai na boto ni sauloa kei nai walewale ni kena laurai na dede gauna era bula tiko kina ena dua na vanua. Na kena vakadeitaki na macala ni vakadidike kece qo ena veidutaitaki ka vakatautauvatataki nai tukutuku ni vakadidike sa qaravi taumada (*historical ecology)* ena dua na gauna salavata kei na kena qaravi vakacakacakataki eso *(experimental)* kei na kena wanonovi matua (*observational studies)* na veika eso sa vakayacori oti.

**Me bulabula na Lase, Veidogo kei na Lewe ni vanua ena veisiga ni mataka:**

Na neitou cakacaka e vakatakila na veiveisau lelevu erau lako curuma na vanua duidui oqo ko Suva kei Savusavu ena gauna sa oti yani. Na duidui kei na rabailevu ni veiveisau erau dui lako curuma e vakatakilai mai na kedrau dui tuvaki, na nodrau dui toba e sa vakatakila na veisau ni kenai tuvaki kei na kena veiyaubula. Era sa raica nai taukei na veiveisau e sotavi ena veimatasawa, na veivunikau era bula ena veimatasawa oqo ka okati talega kina na kena sotavi na veiveisau eso enai tuvaki ni veicakau. O Viti e liu tiko ena vakasama ni maroroi keina taqomaki ni yaubula eso, ka okati kina na veikau ena bati ni uciwai kei na loma ni vanua. Na cakacaka se vakadidike oqo e vakatakila na kena rawa ni vakayagataki na kena vei macala eso. Na kena kilai na veitukutuku makawa sa kumuni taumada ena vakadeitaka nai walewale matau ni kena maroroi se taqomaki nai yaubula eso. Ka kuria na noda rokova ka doka na vanua mamaca kei na waitui e Viti ena vuku ni veisau eso e sotavi ka lavetaki tale eso nai tukutuku ka mapetaki nai walewale matau me vakawataki kina e so na lase kei na veidogodogo bulabula ka okati talega kina na lewenivanua bulabula.

**Na vakavinavinaka e vakagolei:**

We thank SY Phoo, S Cunningham, and J Frair for advice during the initial planning stages of this manuscript. A. Caginitoba and W. Nasilisili provided help on Fijian language translations and nuances. L. McClenachan and A. Cabinitoba gave useful comments on an earlier version of this manuscript. We thank the research librarians at the institutions we obtained charts and photos for this study. Funding was provided by a Franklin Research Grant from the American Philosophical Society to JD.

**Authors’ Contributions:** JD and KNL conceived the idea. KNL and HL collected and analyzed the data. KNL and JD led the writing of the manuscript. JD provided funding. All authors contributed critically to the drafts and gave final approval for publication.

**Data Availability:** Data available at the ESF Digital Commons. Information on the charts included in this analysis is available in the Supporting Material.

**References**

Alongi DM (2014) Carbon Cycling and Storage in Mangrove Forests. Annual Review of Marine Science 6(1):195–219. <https://doi.org/10.1146/annurev-marine-010213-135020>

Ault TR, Johnson CR (1998) Spatially and Temporally Predictable Fish communities on Coral Reefs. Ecological Monographs 68(1):25–50. [https://doi.org/10.1890/0012-9615(1998)068[0025:SATPFO]2.0.CO;2](https://doi.org/10.1890/0012-9615(1998)068%5b0025:SATPFO%5d2.0.CO;2)

Bao K, Drew J (2017) Traditional ecological knowledge, shifting baselines, and conservation of Fijian molluscs. Pac Conserv Biol 23(1):81. <https://doi.org/10.1071/PC16016>

Barrett JW (1935) To Melbourne and Back. Br Med J 1(3882):1147

Bax N, Hayes K, Marshall A, Parry D, Thresher R (2002) Man-made marinas as sheltered islands for alien marine organisms: Establishment and eradication of an alien invasive marine species. In: Turning the tide: the eradication of invasive species. IUCN SSC Invasive Species Specialist Group. IUCN, Gland, Switzerland and Cambridge, UK, p 14

Bellwood DR, Hughes TP (2001) Regional-Scale Assembly Rules and Biodiversity of Coral Reefs. Science 292(5521):1532–1535. <https://doi.org/10.1126/science.1058635>

Bligh W (2013) A Voyage to the South Sea, for the purpose of conveying the bread-fruit tree to the West Indies: In His Majesty’s ship the Bounty, commanded by Lieutenant William Bligh. Cambridge University Press

Bonin MC, Almany GR, Jones GP (2011) Contrasting effects of habitat loss and fragmentation on coral-associated reef fishes. Ecology 92(7):1503–1512. <https://doi.org/10.1890/10-0627.1>

Bromberg KD, Bertness MD (2005) Reconstructing New England salt marsh losses using historical maps. Estuaries 28(6):823–832. <https://doi.org/10.1007/BF02696012>

Bromley C, McGonigle C, Ashton EC, Roberts D (2016) Bad moves: Pros and cons of moving oysters – A case study of global translocations of Ostrea edulis Linnaeus, 1758 (Mollusca: Bivalvia). Ocean & Coastal Management 122:103–115. <https://doi.org/10.1016/j.ocecoaman.2015.12.012>

Brooker RM, Seyfferth AL, Hunter A, Sneed JM, Dixson DL, Hay ME (2020) Human proximity suppresses fish recruitment by altering mangrove-associated odour cues. Scientific Reports 10(1):21091. <https://doi.org/10.1038/s41598-020-77722-7>

Caley MJ, Buckley KA, Jones GP (2001) Separating ecological effects of habitat fragmentation, degradation, and loss on coral commensals. Ecology 82(12):3435–3448. [https://doi.org/10.1890/0012-9658(2001)082[3435:SEEOHF]2.0.CO;2](https://doi.org/10.1890/0012-9658(2001)082%5b3435:SEEOHF%5d2.0.CO;2)

Cameron C, Maharaj A, Kennedy B, Tuiwawa S, Goldwater N, Soapi K, Lovelock CE (2021) Landcover change in mangroves of Fiji: Implications for climate change mitigation and adaptation in the Pacific. Environmental Challenges 2:100018. <https://doi.org/10.1016/j.envc.2020.100018>

Carney J (2017) “The mangrove preserves life”: Habitat of African survival in the Atlantic world. Geographical Review 107(3):433–451. <https://doi.org/10.1111/j.1931-0846.2016.12205.x>

Charan H, N’Yeurt ADR, Iese V, Chopin T (2017) The effect of temperature on the growth of two pest seaweeds in Fiji. 2nd International Conference on Energy, Environment and Climate :7

Cheal AJ, MacNeil MA, Emslie MJ, Sweatman H (2017) The threat to coral reefs from more intense cyclones under climate change. Global Change Biology 23(4):1511–1524. <https://doi.org/10.1111/gcb.13593>

Cochrane E (2017) Ancient Fiji: Melting pot of the Southwest Pacific. Oxford University Press

Costa M, Le Baron N, Tenhunen K, Nephin J, Willis P, Mortimor JP, Dudas S, Rubidge E (2020) Historical distribution of kelp forests on the coast of British Columbia: 1858–1956. Applied Geography 120:102230. <https://doi.org/10.1016/j.apgeog.2020.102230>

Cumming RL, Toscano MA, Lovell ER, Carlson BA, Dulvy NK, Hughes A, Koven JF, Sykes HR, Taylor OJS, Vaughan D (2006) Mass coral bleaching in the Fiji Islands, 2000. :8

Dacks R, Ticktin T, Jupiter SD, Friedlander AM (2020) Investigating the role of fish and fishing in sharing networks to build resilience in coral reef social-ecological systems. Coastal Management 48(3):165–187. <https://doi.org/10.1080/08920753.2020.1747911>

Dana JD (1849) United States Exploring Expedition: During the years 1838, 1839, 1840, 1841, 1842 under the command of Charles Wilkes. Atlas zoophytes. C. Sherman

Davis MT, Newell PF, Quinn NJ (1999) TBT contamination of an artisanal subsistence fishery in Suva harbour, Fiji. Ocean & Coastal Management 42(6):591–601. <https://doi.org/10.1016/S0964-5691(99)00035-6>

Davis WM (1918) The reef-encircled islands of the Pacific. Journal of Geography 17(2):58–68. <https://doi.org/10.1080/00221341808984389>

Duke NC, Meynecke J-O, Dittmann S, Ellison AM, Anger K, Berger U, Cannicci S, Diele K, Ewel KC, Field CD, Koedam N, Lee SY, Marchand C, Nordhaus I, Dahdouh-Guebas F (2007) A World Without Mangroves? Science 317(5834):41b–42b. <https://doi.org/10.1126/science.317.5834.41b>

Feller IC, Whigham DF, O’Neill JP, McKee KL (1999) Effects of nutrient enrichment on within-stand cycling in a mangrove forest. Ecology 80(7):2193–2205. [https://doi.org/10.1890/0012-9658(1999)080[2193:EONEOW]2.0.CO;2](https://doi.org/10.1890/0012-9658(1999)080%5b2193:EONEOW%5d2.0.CO;2)

Friedlander A, Parrish J (1998) Habitat characteristics affecting fish assemblages on a Hawaiian coral reef. Journal of Experimental Marine Biology and Ecology 224:1–30. <https://doi.org/10.1016/S0022-0981(97)00164-0>

Goberdhan L, Kininmonth S (2021) Insights into coral growth rate trends in Fiji. Coral Reefs 40(1):251–266. <https://doi.org/10.1007/s00338-020-02037-y>

Granek EF, Ruttenberg BI (2007) Protective capacity of mangroves during tropical storms: a case study from ‘Wilma’ and ‘Gamma’ in Belize. Marine Ecology Progress Series 343:101–105.

Goldberg J, Wilkinson C (2004) 1. Global threats to coral reefs: coral bleaching, global climate change, disease, predator plagues, and invasive species. Status of Coral Reefs of the World: 2004 :26

Graci S, Vliet LV (2020) Examining stakeholder perceptions towards sustainable tourism in an island destination. The Case of Savusavu, Fiji. Tourism Planning & Development 17(1):62–81. <https://doi.org/10.1080/21568316.2019.1657933>

Hesselbarth M, Sciaini M, Wiengand K, Nowosad J (2019) landscapemetrics: an open‐source R tool to calculate landscape metrics. Ecography 42:1648-1657 (ver. 0)

Hutchings PA, Hilliard RW, Coles SL (Stephen L (2002) Species introductions and potential for marine pest invasions into tropical marine communities, with special reference to the Indo-Pacific. Pacific Science 56(2):223–233. <https://doi.org/10.1353/psc.2002.0017>

Inskip TWH (1937) Strategic importance of Pacific Islands report by New Zealand Government. In: Institute of Latin American Studies. <https://sas-space.sas.ac.uk/8288/>. Accessed 10 Feb 2021

Jenny B, Hurni L (2011) Studying cartographic heritage: Analysis and visualization of geometric distortions. Computers & Graphics 35(2):402–411. <https://doi.org/10.1016/j.cag.2011.01.005>

Klein ES, Thurstan RH (2016) Acknowledging long-term ecological change: The problem of Shifting Baselines. In: Schwerdtner Máñez K, Poulsen B (eds) Perspectives on Oceans Past. Springer Netherlands, Dordrecht, pp 11–29. <https://doi.org/10.1007/978-94-017-7496-3_2>

Knowlton N (2001) Coral reef biodiversity--Habitat size matters. Science 292(5521):1493–1495. <https://doi.org/10.1126/science.1061690>

Lal PN (1990) Conservation or conversion of mangroves in Fiji: an ecological economic analysis. Environment and Policy Institute, East-West Center, Honolulu, Hawaii

Lal R, Kininmonth S, N’Yeurt ADR, Riley RH, Rico C (2018) The effects of a stressed inshore urban reef on coral recruitment in Suva Harbour, Fiji. Ecology and Evolution 8(23):11842–11856. <https://doi.org/10.1002/ece3.4641>

Lukas MC (2014) Cartographic Reconstruction of Historical Environmental Change. Cartographic Perspectives (78):5–24. <https://doi.org/10.14714/CP78.1218>

McClenachan L, Cooper AB, McKenzie MG, Drew JA (2015) The importance of surprising results and best practices in Historical Ecology. BioScience 65(9):932–939. <https://doi.org/10.1093/biosci/biv100>

McClenachan L, O’Connor G, Neal BP, Pandolfi JM, Jackson JBC (2017) Ghost reefs: Nautical charts document large spatial scale of coral reef loss over 240 years. Science Advances 3(9):e1603155. <https://doi.org/10.1126/sciadv.1603155>

Mumby PJ (2006) Connectivity of reef fish between mangroves and coral reefs: Algorithms for the design of marine reserves at seascape scales. Biological Conservation 128(2):215–222. <https://doi.org/10.1016/j.biocon.2005.09.042>

Munday PL (2004) Habitat loss, resource specialization, and extinction on coral reefs. Global Change Biology 10(10):1642–1647. <https://doi.org/10.1111/j.1365-2486.2004.00839.x>

Naidu SD, Morrison RJ (1994) Contamination of Suva harbour, Fiji. Marine Pollution Bulletin 29(1):126–130. <https://doi.org/10.1016/0025-326X(94)90436-7>

O’Garra T (2009) Bequest values for marine resources: How important for Indigenous communities in less-developed economies? Environ Resource Econ 44:179–202. <https://doi.org/10.1007/s10640-009-9279-3>

Palandro DA, Andréfouët S, Hu C, Hallock P, Müller-Karger FE, Dustan P, Callahan MK, Kranenburg C, Beaver CR (2008) Quantification of two decades of shallow-water coral reef habitat decline in the Florida Keys National Marine Sanctuary using Landsat data (1984–2002). Remote Sensing of Environment 112(8):3388–3399. <https://doi.org/10.1016/j.rse.2008.02.015>

Pauly D (1995) Anecdotes and the shifting baseline syndrome of fisheries. Trends in Ecology & Evolution 10(10):430. <https://doi.org/10.1016/S0169-5347(00)89171-5>

Penn N (1983) The environmental consequences and management of coral sand dredging in the Suva region,. Diss University College of Swansea :308

Prasad RD, Raturi A (2019) Low carbon alternatives and their implications for Fiji’s electricity sector. Utilities Policy 56:1–19. <https://doi.org/10.1016/j.jup.2018.10.007>

Pratchett MS, Hoey AS, Wilson SK, Messmer V, Graham NAJ (2011) Changes in biodiversity and functioning of reef fish assemblages following coral bleaching and coral loss. Diversity 3(3):424–452. <https://doi.org/10.3390/d3030424>

R Core Team (2020) R: A language and environment for statistical computing. R Foundation for Statistical Computing, Vienna, Austria. URL https://www.R-project.org/

Rhodes KL, Hernandez-Ortiz DX, Cuetos-Bueno J, Ioanis M, Washington W, Ladore R (2018) A 10-year comparison of the Pohnpei, Micronesia, commercial inshore fishery reveals an increasingly unsustainable fishery. Fisheries Research 204:156–164. <https://doi.org/10.1016/j.fishres.2018.02.017>

Sambrook K, Jones GP, Bonin MC (2016) Life on the edge: Coral reef fishes exhibit strong responses to a habitat boundary. Marine Ecology Progress Series 561:203–215

Sangha KK, Maynard S, Pearson J, Dobriyal P, Badola R, Hussain SA (2019) Recognising the role of local and Indigenous communities in managing natural resources for the greater public benefit: Case studies from Asia and Oceania region. Ecosystem Services 39:100991. <https://doi.org/10.1016/j.ecoser.2019.100991>

Seemann B (1862) Viti: An account of a government mission to the Vitian or Fijian Islands, in the years 1860-61. Macmillan & Company

Smith JW (2013) The bound[less] sea: Wilderness and the United States Exploring Expedition in the Fiji Islands. Environmental History 18(4):710–737. <https://doi.org/10.1093/envhis/emt067>

Spalding MD, Brown BE (2015) Warm-water coral reefs and climate change. Science 350(6262):769–771. <https://doi.org/10.1126/science.aad0349>

Sparling CH (1923) A short cruise in the South Seas. The Geographical Teacher 12(1):60–64

Sulu R, Kumar L, Hay C, Pickering T (2003) Kappaphycus seaweed in the Pacific: review of introductions and field testing proposed quarantine protocols. Noumea: Secretariat of the Pacific Community :85

Thampanya U, Vermaat JE, Sinsakul S, Panapitukkul N (2006) Coastal erosion and mangrove progradation of Southern Thailand. Estuarine, Coastal and Shelf Science 68(1):75–85. <https://doi.org/10.1016/j.ecss.2006.01.011>

Thurstan RH, McClenachan L, Crowder LB, Drew JA, Kittinger JN, Levin PS, Roberts CM, Pandolfi JM (2015) Filling historical data gaps to foster solutions in marine conservation. Ocean & Coastal Management 115:31–40. <https://doi.org/10.1016/j.ocecoaman.2015.04.019>

Vellend M, Brown CD, Kharouba HM, McCune JL, Myers‐Smith IH (2013) Historical ecology: Using unconventional data sources to test for effects of global environmental change. American Journal of Botany 100(7):1294–1305. <https://doi.org/10.3732/ajb.1200503>

Ward RG (1959) The Population of Fiji. Geographical Review 49(3):322–341. <https://doi.org/10.2307/211910>

Whipple AA, Grossinger RM, Davis FW (2011) Shifting Baselines in a California Oak Savanna: Nineteenth century data to inform restoration scenarios. Restoration Ecology 19(101):88–101. <https://doi.org/10.1111/j.1526-100X.2009.00633.x>

Wilkes C (1845) Narrative of the United States’ Exploring Expedition: During the years 1838, 1839, 1840, 1841, 1842. Whittaker

**Supporting Information**

Copyright details and links to the source are available for all images included in this manuscript (Appendix S1) are available online. The authors are solely responsible for the content and functionality of these materials. Queries (other than absence of the material) should be directed to the corresponding author.

**Figure Legends**

Fig. 1. Timeline of relevant historical events occurring within the time analyzed in this study. Top row left to right, historical photos show Suva Harbor in 1890 (Charles Kerry, National Library of Australia), 1914 (Australian War Museum), and 1956 (Australian War Museum) and bottom row, left to right shows a postcard of Savusavu in 1888 (State Library Victoria) and a photo from 1966 (Michael Terry, National Library of Australia). Grey dashes show charts analyzed in this study. See Supporting Information for more photo source information.

Fig. 2. Locations of Suva and Savusavu in Fiji, with boxes showing the approximate extent of charts used in this project.

Fig. 3. Clipping of a 1945 Suva chart, acquired from State Library of New South Wales (Table 1). Color of original chart has been edited to grayscale to highlight features of interest. In clipping, coral is represented by hatched coastline on the original chart and highlighted in orange, mangrove is represented by tree symbols and curled lines on the original and highlighted in green, while hardened shorelines is represented by dark grey lines and shapes and highlighted in yellow.

Fig. 4. Area (top; a, c) and Perimeter (bottom; b, d) of coral reefs in Suva (left; a, b) and Savusavu (right; c, d). Earliest charts, which depicted less detail than the latest charts, are shown as X. Gray backgrounds identify the location but are not to scale.

Fig. 5. Number of reef habitat patches in Savusavu, in the wider view (left) and nearshore (right). Inset box shows the location of nearshore Savusavu on the wider chart. Earliest charts, which depicted less detail than the latest charts, are shown as X. Gray backgrounds identify the location but are not to scale.

Fig. 6. Coastline use in Suva (top; a) and Savusavu (bottom; b, c). In Savusavu, mangroves represented a smaller part of the coastline closer to town (b) than over the entire bay (c). Coastline is occupied by either Urban infrastructure, Mangrove forests, or Other, natural or agricultural landscapes. Gray backgrounds identify the location but are not to scale.

**Figures**


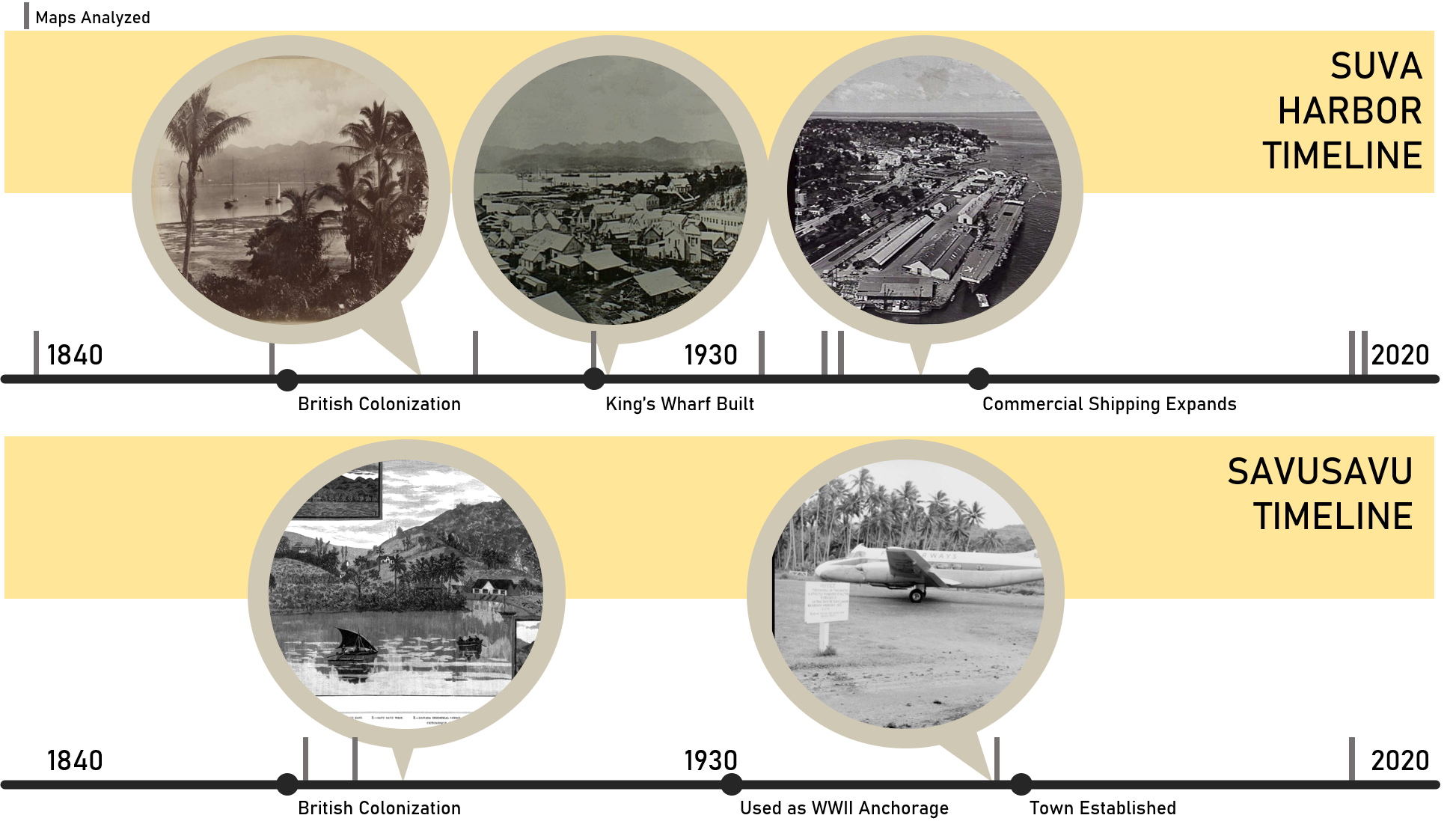


Fig. 1. Timeline of relevant historical events occurring within the time analyzed in this study. Top row left to right, historical photos show Suva Harbor in 1890 (Charles Kerry, National Library of Australia), 1914 (Australian War Museum), and 1956 (Australian War Museum) and bottom row, left to right shows a postcard of Savusavu in 1888 (State Library Victoria) and a photo from 1966 (Michael Terry, National Library of Australia). Grey dashes show charts analyzed in this study. See Supporting Information for more photo source information.


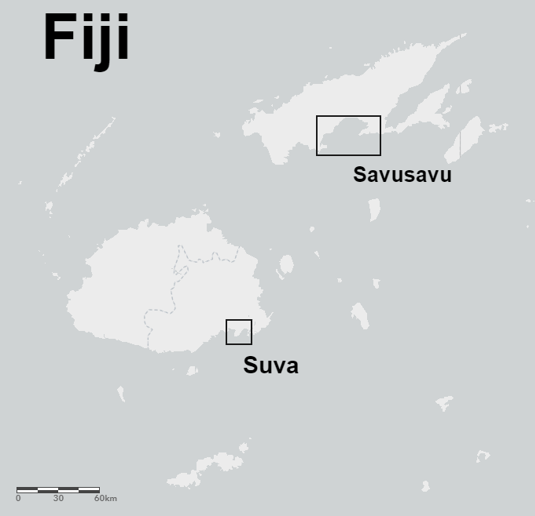


Fig. 2. Locations of Suva and Savusavu in Fiji, with boxes showing the approximate extent of charts used in this project.


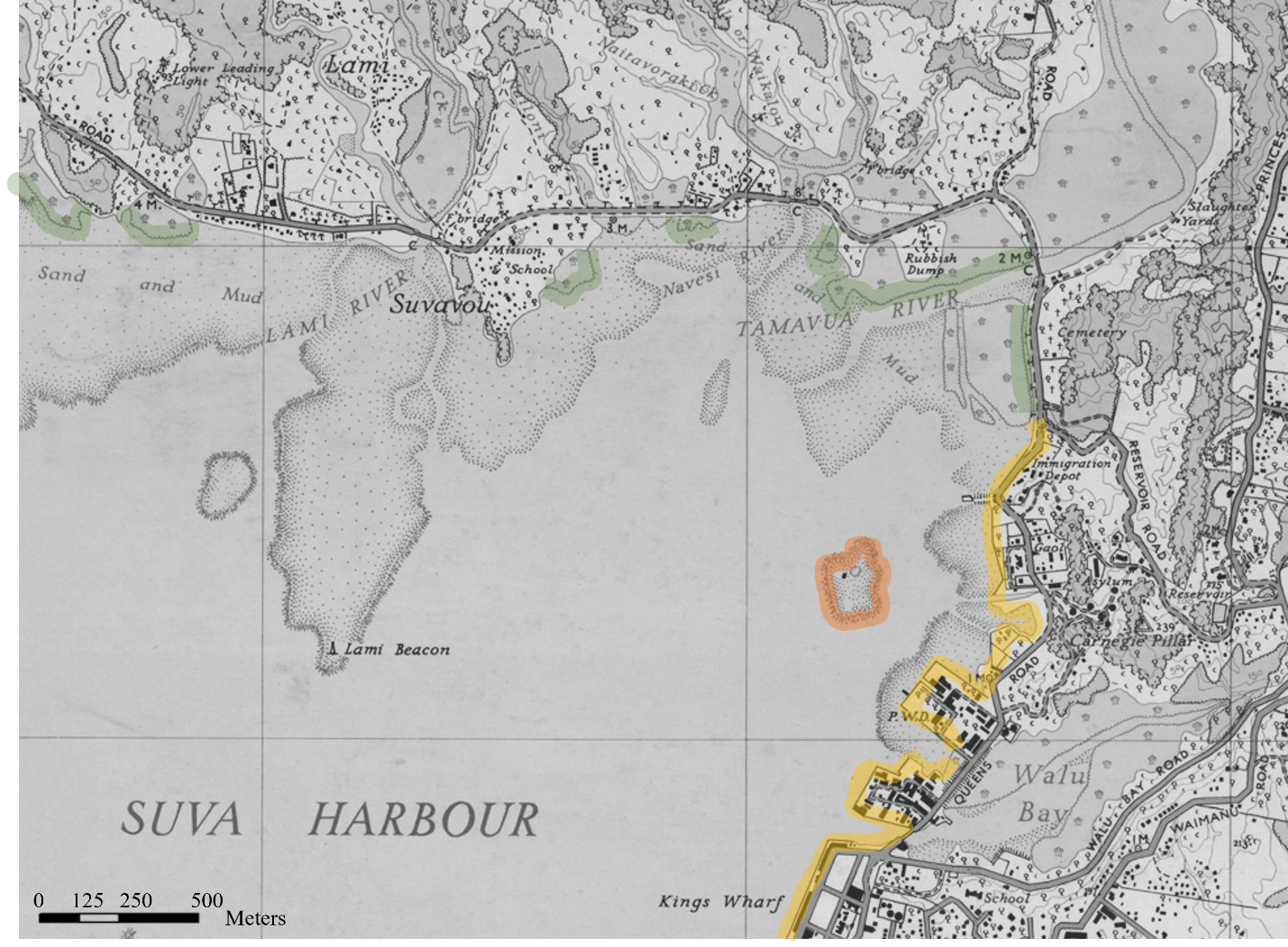


Fig. 3. Clipping of a 1945 Suva chart, acquired from State Library of New South Wales (Table 1). Color of original chart has been edited to grayscale to highlight features of interest. In clipping, coral is represented by hatched coastline on the original chart and highlighted in orange, mangrove is represented by tree symbols and curled lines on the original and highlighted in green, while hardened shorelines is represented by dark grey lines and shapes and highlighted in yellow.


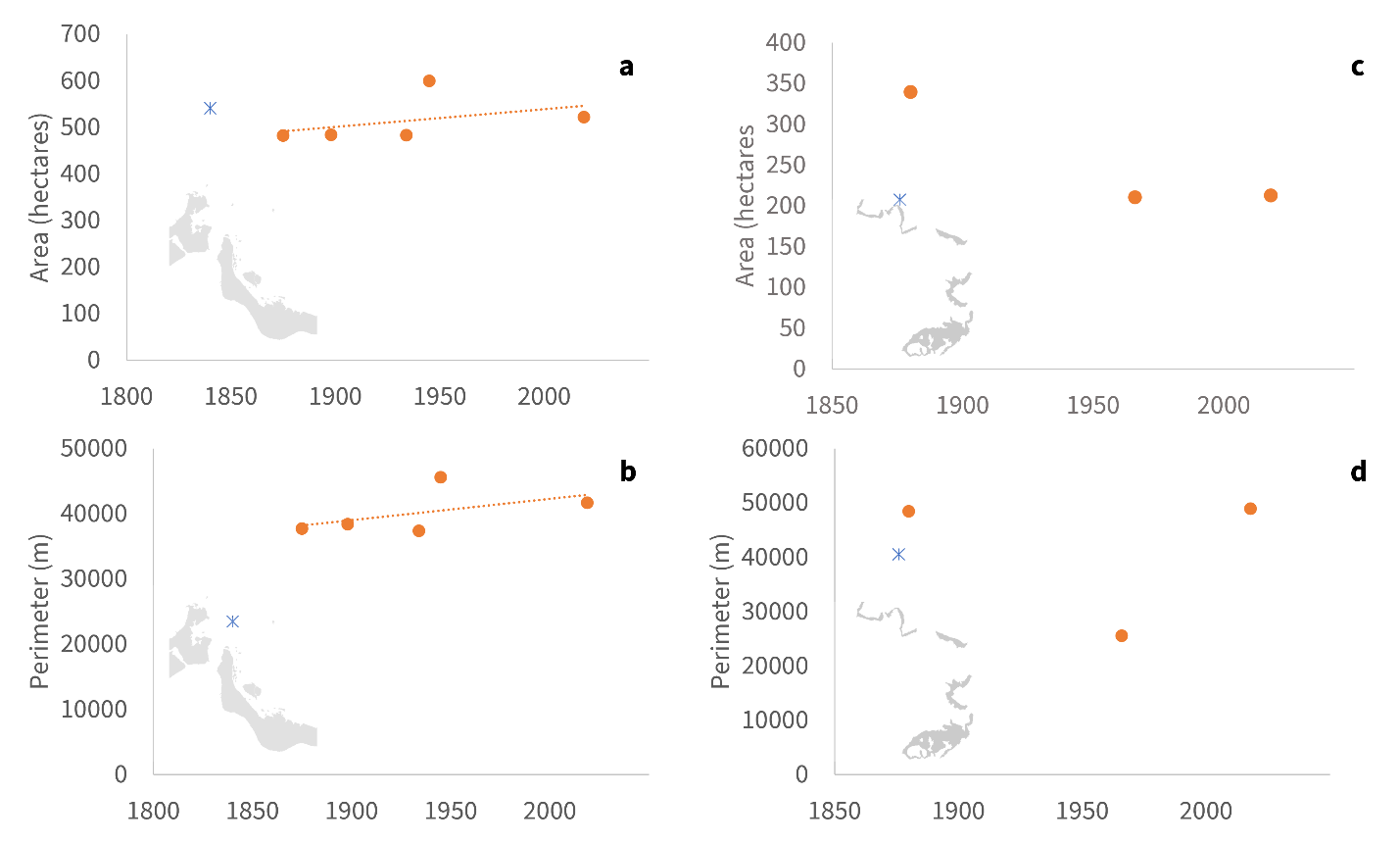


Fig. 4. Area (top; a, c) and Perimeter (bottom; b, d) of coral reefs in Suva (left; a, b) and Savusavu (right; c, d). Earliest charts, which depicted less detail than the latest charts, are shown as X. Gray backgrounds identify the location but are not to scale.


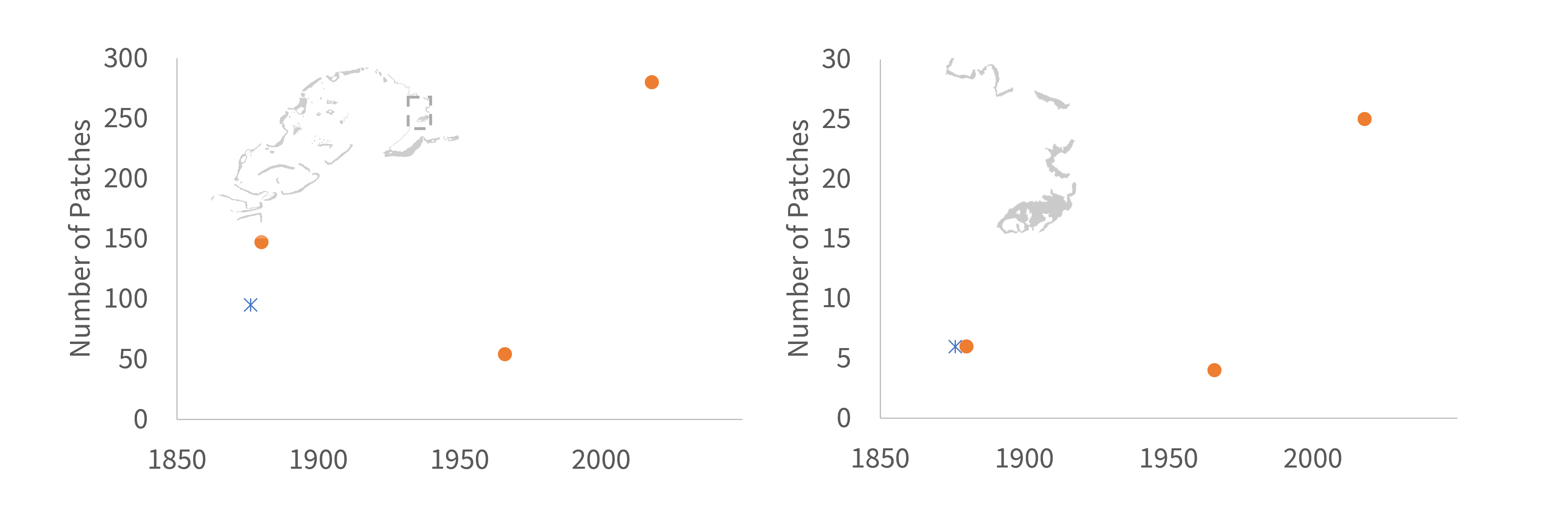


Fig. 5. Number of reef habitat patches in Savusavu, in the wider view (left) and nearshore (right). Inset box shows the location of nearshore Savusavu on the wider chart. Earliest charts, which depicted less detail than the latest charts, are shown as X. Gray backgrounds identify the location but are not to scale.


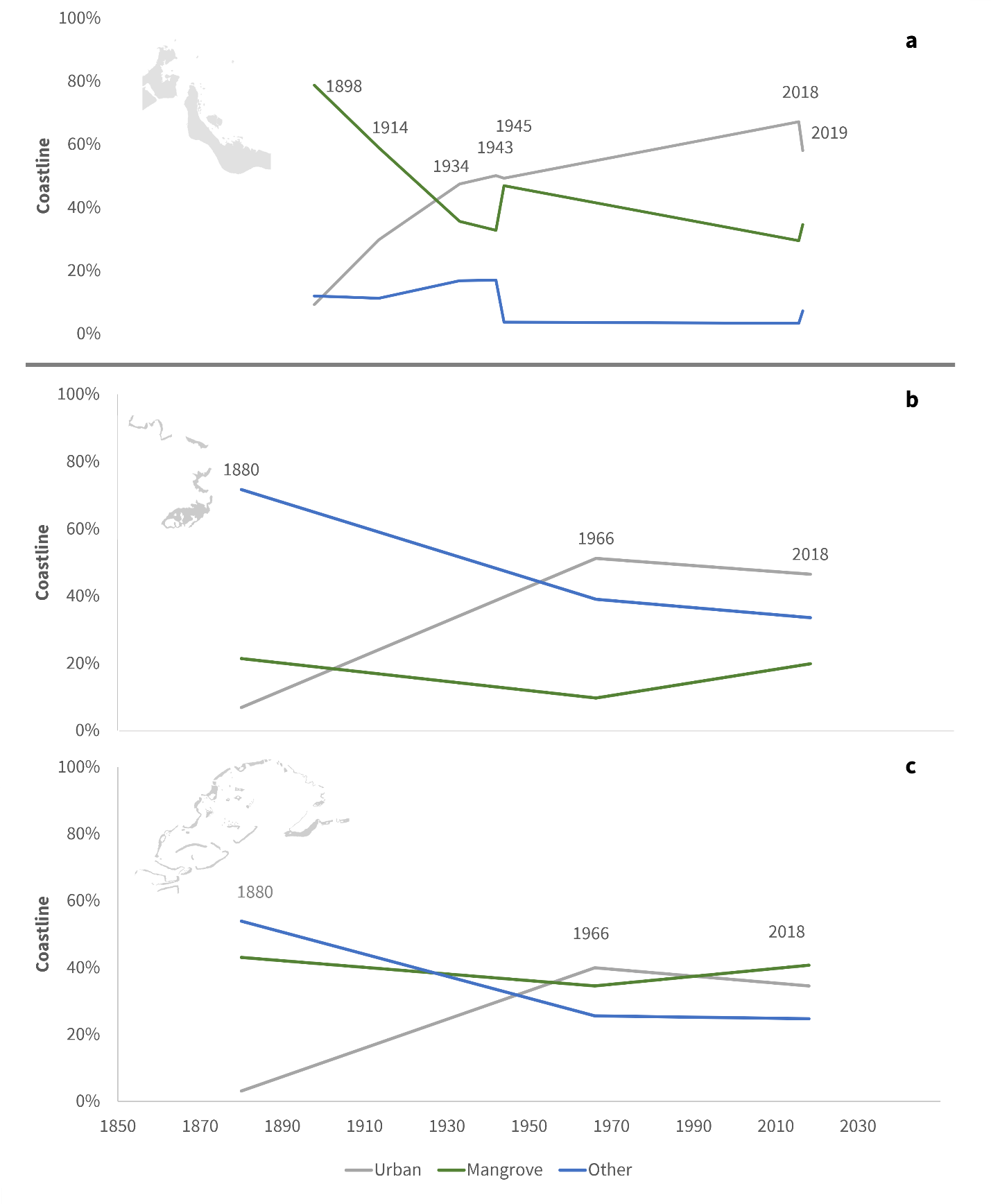


Fig. 6. Coastline use in Suva (top; a) and Savusavu (bottom; b, c). In Savusavu, mangroves represented a smaller part of the coastline closer to town (b) than over the entire bay (c). Coastline is occupied by either Urban infrastructure, Mangrove forests, or Other, natural or agricultural landscapes. Gray backgrounds identify the location but are not to scale.
